# Predictors of disease burden in patients with untreated transthyretin amyloid cardiomyopathy and their caregivers: a *post hoc* analysis of an international survey

**DOI:** 10.3389/fcvm.2025.1595797

**Published:** 2025-06-09

**Authors:** Francesco Cappelli, Lucia Ponti, Kristen Hsu, Thibaud Damy, Eduardo Villacorta, Nicolas Verheyen, Denis Keohane, Ronnie Wang, Monica Ines, Nisith Kumar, Carmen Munteanu

**Affiliations:** ^1^ Tuscan Regional Amyloidosis Referral Centre, Careggi University Hospital, Florence, Italy; ^2^ University of Urbino, Urbino, Italy; ^3^ Amyloidosis Research Consortium, Newton, MA, United States; ^4^ Henri Mondor Hospital, Paris, France; ^5^ Complejo Asistencial Universitario de Salamanca, Salamanca, Spain; ^6^ Medical University of Graz, Graz, Austria; ^7^ Pfizer Inc., New York, NY, United States; ^8^ Pfizer Inc., Groton, CT, United States; ^9^ Pfizer Inc., Porto Salvo, Portugal

**Keywords:** amyloidosis, symptoms, care burden, heart failure, patient-reported outcome measures, quality of life

## Abstract

**Introduction:**

Transthyretin amyloid cardiomyopathy (ATTR-CM) is a progressive condition with debilitating symptoms. The self-reported burden of ATTR-CM on patients and their caregivers was previously evaluated in an international, multicenter, real-world survey study.

**Methods:**

This *post hoc* analysis used univariate and multivariate models to evaluate survey items as predictors of ATTR-CM burden. The final multivariate models were optimized using forward selection and CV Press criteria with 8-fold cross-validation to include only the best predictors. Hierarchical linear regression analyses were used to explore potential moderators of the relationship between patient health status and caregiver burden.

**Results:**

The original survey included 208 patients with ATTR-CM, naïve to disease-modifying treatment, and their unpaid primary caregivers from international amyloidosis centers of excellence in 7 countries between July 2021 and August 2022. Most patients were male (86%), elderly (median age, 81 years), and had untreated wild-type ATTR-CM (91% of 155 with genetic testing). Patients reported fair to good health status overall [Kansas City Cardiomyopathy Questionnaire Overall Summary (KCCQ-OS) median score, 68]. Most (60%) of the 199 patients with a New York Heart Association (NYHA) classification were class II (18% class I; 22% class III). Optimized multivariate models for several measures found symptomatic heart failure (NYHA class II or III), having “weakness, especially in the legs,” older age, and female sex, were independent predictors of higher patient-reported burden. The majority of caregivers were female (85%) and the spouse (59%) or adult child (37%) of the patient. The median duration of caregiving was 1.5 years. In the final optimized multivariate model, only the patient's KCCQ-OS score was a significant predictor of caregiver burden. This relationship was not clinically moderated by other patient or caregiver variables.

**Conclusions:**

Our analysis showed that heart failure symptoms, weakness, especially in the legs, older age, and female sex, are independent predictors of higher disease burden in patients with ATTR-CM. A higher caregiver burden was best predicted by poorer health status in the patient, even in the presence of potential moderators. Implementing strategies to reduce the physical symptoms experienced by patients with ATTR-CM may help to reduce their burden, and that experienced by caregivers.

## 1 Introduction

Transthyretin amyloid cardiomyopathy (ATTR-CM) is a progressive condition that can cause debilitating symptoms largely associated with heart failure (1–3). Several studies have evaluated the burden of ATTR-CM in patients and their caregivers, but usually utilizing a small number of established measures, in single centers, or as a minor part of a larger study (1, 4–15). The burden of untreated ATTR-CM on patients and their caregivers was recently evaluated in an international, multicenter, real-world survey. This study included participants from international centers and measured multiple aspects of burden (16). Most patients in the study had recently diagnosed symptomatic wild-type ATTR-CM, and all were naïve to disease-modifying treatment. Caregivers were unpaid and were usually the female spouse or adult child of the patient. An important feature of this study was that survey responses for patients and their caregivers were paired, enhancing the ability to evaluate factors contributing to burden. The study found that both patients and their caregivers felt a physical and mental burden from ATTR-CM, and this appeared to be higher when the patient had more severe heart failure symptoms (16).

Identifying predictors of ATTR-CM burden could help improve the therapeutic journey for patients and guide approaches to limit the burden on caregivers. This *post hoc* analysis aimed to identify predictors of burden on patients with untreated ATTR-CM and their caregivers using data from the recent international real-world survey (16).

## 2 Materials and methods

Methodology and primary findings from the international, multicenter, real-world survey have been published (16). All patients and caregivers provided informed consent to participate in the study. Consent forms were approved by the institutional review board or independent ethics committee at each site before use.

**Figure 1:**
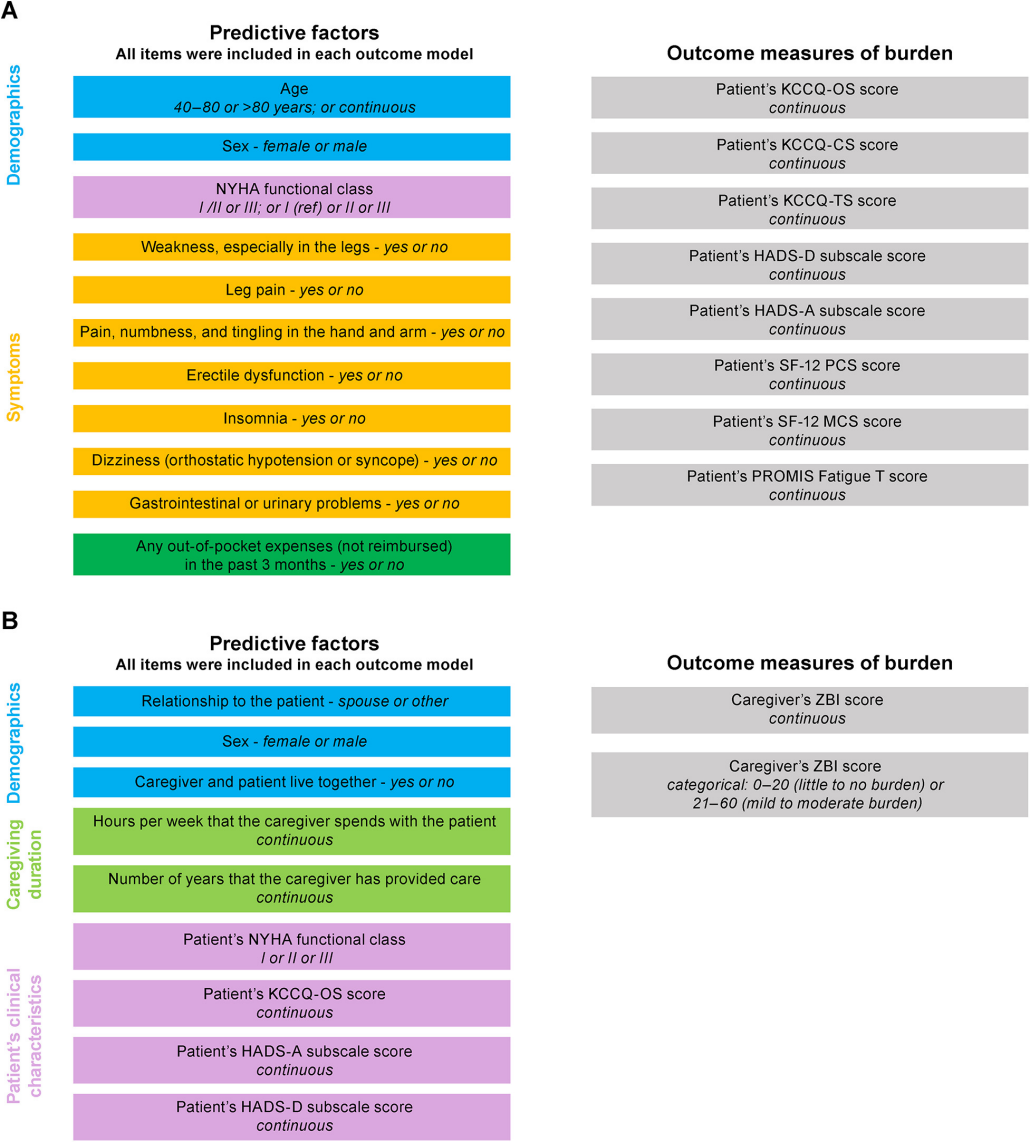
Items included in the **(A)** patient and **(B)** caregiver models. CS, clinical summary; HADS-A, Hospital Anxiety and Depression Scale anxiety; HADS-D, Hospital Anxiety and Depression Scale depression; KCCQ, Kansas City Cardiomyopathy Questionnaire; MCS, mental component summary; NYHA, New York Heart Association; OS, overall summary; PCS, physical component summary; PROMIS, Patient-Reported Outcomes Measurement Information System; SF-12; 12-item Short Form Health Survey; TS, total symptoms; ZBI, Zarit Burden Interview.

This *post hoc* analysis used univariate and multivariate models to evaluate selected survey items as potential predictors of patient or caregiver burden. Items from the survey selected for initial inclusion were chosen by the authors based on their experience in the disease area and overview of the complete survey data. The included items evaluated as predictors of patient burden are shown in Figure 1A. Of these, the patient-reported survey responses were used to calculate their out-of-pocket expenses; 23-item Kansas City Cardiomyopathy Questionnaire (KCCQ) Overall Summary (OS), Clinical Summary (CS), and Total Symptoms (TS) scores (17); Hospital Anxiety and Depression Scale depression (HADS-D) and anxiety (HADS-A) subscale scores (18); 12-item Short Form Health Survey (SF-12) V2 Physical Component Summary (PCS) and Mental Component Summary (MCS) scores (19–22); and Patient-Reported Outcomes Measurement Information System (PROMIS) Fatigue Severity Short Form 7a V1.0 score (23, 24). Patient age, sex, New York Heart Association (NYHA) functional classification, and current symptoms were reported from their medical records by the investigator. Of relevance, several measures have previously been associated with prognosis in patients with ATTR-CM, including NYHA class and KCCQ-OS score (25, 26).

Items evaluated as predictors of caregiver burden are shown in Figure 1B. Of these, caregiver survey responses were used to define their relationship to the patient, if they lived with the patient, their duration of caregiving, and to calculate their 22-item Zarit Burden Interview (ZBI) score (27). The KCCQ-OS, HADS-A, and HADS-D scores for the patient that they cared for were derived as described for the patient models. The caregiver's sex and the NYHA class of the patient they cared for were provided by the investigator.

Unadjusted univariate associations examined the relationship between each predictor and each outcome measure of burden. The strength of these relationships was tested using a *t* test, linear or ordinal logistic regression, and Pearson correlation coefficient, as appropriate. The full multivariate models examined the relationship between multiple predictors and a single outcome measure of burden. The full models included all the predictors in this study. The optimized models identified the best set of predictors for each outcome measure of burden using forward selection (i.e., adding predictors to the no effect model). The final optimized models were validated using CV Press criteria within the 8-fold cross-validation procedure. To do this, data were randomly split into training sets and test sets. Each of the 8 validation procedures was performed by using the multivariate model obtained from forward selection on the 7/8 parts of data, or as the training set. The fitted model was then used to compute the predicted residual sum of squares on the 1/8 parts of the data, or as the test set. This was repeated 8 times. The sum of the 8 predicted residual sum of squares was obtained to estimate the prediction error denoted by the CV Press score. The model with the candidate predictors that yielded the smallest CV Press score was selected as the final optimal model. This approach allowed us to test the validity of our model 8 times, leading to a more accurate result than if we had tested it fewer times. This assessment method was robust and produced an accurate estimate of model performance.

A moderator is something that changes the nature of the relationship between 2 others. In the sensitivity analysis, we examined if selected survey items (covariates) could influence the relationship identified between the patient's continuous KCCQ-OS score and the caregiver's continuous ZBI score. Covariates tested as potential moderators were patient-reported HADS-A, HADS-D, PROMIS Fatigue, SF-12 PCS, and SF-12 MCS scores, the patient's NYHA functional class, severity of specific symptoms over the last 7 days (rated 0–10 with increasing severity), and presence of a range of symptoms that can be associated with ATTR-CM; and caregiver-reported age, relationship to patient, sex, hours per week that the caregiver spends with the patient, number of years the caregiver has provided care, if the patient and caregiver live together, HADS-A, HADS-D, PROMIS Fatigue, SF-12 PCS, and SF-12 MCS scores, and whether the patient needs help with a range of daily activities. Baseline averages for these survey items have been published (16). Similar to the approach used in Ponti et al. (28), hierarchical linear regression analyses were used to explore the moderator effect of each survey item in 3 steps. In the first step, the relationship between the patient's KCCQ-OS score and the caregiver's ZBI score was calculated. The second and third steps were then conducted separately for each potential moderator. In the second step, we looked at the effect of adding the covariate to the model. In the third step, we looked at the effect of further adding the 2-way interaction between the covariate and continuous KCCQ-OS. A significant moderator was identified where (1) the interaction term showed a statistically significant relationship with continuous ZBI score (*P* < 0.05) and (2) there was a clinically significant change in the regression coefficient when the covariate was included in the model.

## 3 Results

### 3.1 Patient and caregiver populations

Overall, 208 patients and 208 caregivers were enrolled in the original study, though not all provided data on each survey item (16). Ninety-five patient and caregiver pairs were in Italy, 34 in France, 31 in Spain, 17 in Australia, 15 in Austria, 10 in Canada, and 6 in Russia (16). The patient population was generally male (86%; 14% were female) with a median age of 81 years (range, 46–90 years) (16). Of the 155 patients with genetic testing data, 91% had wild-type ATTR-CM (16). Among the 207 patients with data, the median time since diagnosis was 0.5 years (interquartile range, 0.1–1.3 years). A summary of patient characteristics directly relevant to this analysis is shown in Table 1 and full data are available in the published primary analysis (16). At the time of the survey, most (60%) of the 199 patients with an available NYHA functional classification were class II; 22% were class III and 18% were class I. Symptoms included in this analysis currently affected 12%–20% of patients. Out-of-pocket expenses (not reimbursed) were incurred in the last 3 months by 39% of the 199 patients with reported data. Median KCCQ-OS, -CS, and -TS scores indicated a fair to good health status, whereas median HADS-A and HADS-D subscale scores did not indicate widespread clinically relevant anxiety or depression. Median SF-12 PCS and MCS scores were slightly below published norms for the general population aged ≥75 years [50th percentile scores, 39 and 54, respectively (20)]. The median PROMIS Fatigue T score indicated similar fatigue to that of the general population [norm of 50 (23, 24)].

**Table 1:** Patient characteristics.

Characteristic	All patients *N* = 208
Age, yrs	
Mean (SD)	80 (7.0)
Median (min, max)	81 (46, 90)
Sex, *n* (%)	
Male	179 (86.1)
Female	29 (13.9)
NYHA functional class, *n* (%)	*n* = 199
I	36 (18.1)
II	120 (60.3)
III	43 (21.6)
IV	0
Patients with current symptoms, *n* (%)	
Weakness, especially in the legs	41 (19.7)
Leg pain	28 (13.5)
Pain, numbness, and tingling in the hand and arm	25 (12.0)
Erectile dysfunction	30 (14.4)
Insomnia	29 (13.9)
Dizziness (orthostatic hypotension or syncope)	33 (15.9)
Gastrointestinal or urinary symptoms	36 (17.3)
Out-of-pocket expenses (not reimbursed) in the past 3 months, *n* (%)	78 (39.2) [*n* = 199]
KCCQ score, median (IQR)	
OS	68 (46.4, 84.4) [*n* = 183]
CS	69 (49.5, 87.5) [*n* = 202]
TS	75 (52.1, 91.7) [*n* = 208]
HADS subscale score, median (IQR)	
HADS-A	5 (3.0, 8.0) [*n* = 205]
HADS-D	6 (3.0, 10.0) [*n* = 204]
SF-12 score, median (IQR)	
PCS	36 (27.5, 43.5) [*n* = 206]
MCS	47 (39.4, 54.4) [*n* = 206]
PROMIS fatigue T score, mean (SD)	51 (9.4) [*n* = 203]

CS, clinical summary; HADS, Hospital and Depression Scale; HADS-A, Hospital and Depression Scale anxiety; HADS-D, Hospital Anxiety and Depression Scale depression; IQR, interquartile range; KCCQ, Kansas City Cardiomyopathy Questionnaire; max, maximum; MCS, mental component summary; min, minimum; NYHA, New York Heart Association; OS, overall summary; PCS, physical component summary; PROMIS, Patient-Reported Outcomes Measurement Information System; SD, standard deviation; SF-12, 12-item Short Form Health Survey; TS, total symptoms.

Some sections of this table are reproduced from Ponti L, Hsu K, Damy T, Villacorta E, Verheyen N, Keohane D, et al. Burden of untreated transthyretin amyloid cardiomyopathy on patients and their caregivers by disease severity: results from a multicenter, non-interventional, real-world study. *Front Cardiovasc Med* (2023) 10:1238843. doi: 10.3389/fcvm.2023.1238843. The source is licensed under a CC BY license. © 2023 Ponti, Hsu, Damy, Villacorta, Verheyen, Keohane, Wang, Ines, Kumar, Munteanu and Cappelli.

Survey items evaluated as potential predictive variables for patient burden included age, sex, NYHA class, current symptoms of “weakness, especially in the legs,” “leg pain,” “pain numbness, and tingling in the hand and arm,” “erectile dysfunction,” “insomnia,” “dizziness (orthostatic hypotension or syncope),” “gastrointestinal or urinary problems,” and any out-of-pocket expenses (not reimbursed) in the past 3 months (Figure 1). Outcome measures of burden were KCCQ-OS, -CS, and -TS; HADS-A and HADS-D; SF-12 PCS and SF-12 MCS; and PROMIS Fatigue T scores.

A summary of characteristics for the caregiver population directly relevant to this analysis is shown in Table 2 and full data are available in the published primary analysis (16). The caregiver population in the original study was generally female (85%), and the spouse (59%) or adult child (37%) of the patient. Median age was 68 (range, 32–88) years, median duration of caregiving was 1.5 years, and 66% lived in the same house as the patient. Caregivers spent a median of 71 h per week with the patient. In the ZBI, 35% of caregivers reported at least a mild to moderate burden of care, and none reported a severe burden. The survey items evaluated as potential predictive variables for caregiver burden included their relationship to the patient, their sex, if they lived with the patient, hours spent with the patient per week, the number of years that they had been providing care to the patient, and the patient's NYHA functional class, KCCQ-OS score, HADS-A, and HADS-D scores (Figure 1). Outcome measures of burden were continuous and categorical ZBI scores.

**Table 2:** Caregiver characteristics.

Characteristic	All caregivers *N* = 208
Age, yrs	
Mean (SD)	66 (12.2)
Median (min, max)	68 (32, 88)
Sex, *n* (%)	
Male	32 (15.4)
Female	176 (84.6)
Relationship to the patient, *n* (%)	
Spouse	122 (58.7)
Adult child	77 (37.0)
Parent	2 (1.0)
Sibling	1 (0.5)
Other	6 (2.9)
Caregiver and patient live in the same house, *n* (%)	138 (66.3)
Number of hours that the caregiver and patient spend together in a week, median (IQR)	71 (14.0, 168.0) [*n* = 196]
Number of years that the caregiver has provided care to the patient, median (IQR)	1.5 (0.0, 3.0) [*n* = 166]
ZBI score	
Median (IQR)	13 (3.0, 24.0)
Categorical burden by score, *n* (%)	
Little or none (0–20)	134 (64.4)
Mild to moderate (21–40)	63 (30.3)
Moderate to severe (41–60)	11 (5.3)
Severe (61–88)	0

IQR, interquartile range; max, maximum; min, minimum; SD, standard deviation; ZBI, Zarit Burden Interview.

Some sections of this table are reproduced from Ponti L, Hsu K, Damy T, Villacorta E, Verheyen N, Keohane D, et al. Burden of untreated transthyretin amyloid cardiomyopathy on patients and their caregivers by disease severity: results from a multicenter, non-interventional, real-world study. *Front Cardiovasc Med* (2023) 10:1238843. doi: 10.3389/fcvm.2023.1238843. The source is licensed under a CC BY license. © 2023 Ponti, Hsu, Damy, Villacorta, Verheyen, Keohane, Wang, Ines, Kumar, Munteanu and Cappelli.

### 3.2 Predictors of burden in patients

In the unadjusted univariate models, NYHA class and the symptom “weakness, especially in the legs” were independently associated with all outcome measures of burden (*P* < 0.05; Supplementary Table S1).

In the full multivariate models, which included all predictors, higher NYHA class (III vs. I; II vs. I) was a significant predictor of higher burden in all outcome measures (Supplementary Table S2). The patient-reported symptom “weakness, especially in the legs” was a significant predictor of higher burden as assessed by KCCQ-OS, -CS, and -TS; SF-12 PCS and MCS; and PROMIS Fatigue scores. Female sex was a significant predictor of higher burden as assessed by KCCQ-OS, HADS-A, HADS-D, and SF-12 MCS scores. Older age (either continuous or being >80 vs. 40–80 years old) was a significant predictor of higher burden as assessed by KCCQ-OS, -CS, and -TS; HADS-D; SF-12 PCS; and PROMIS Fatigue scores.

**Figure 2:**
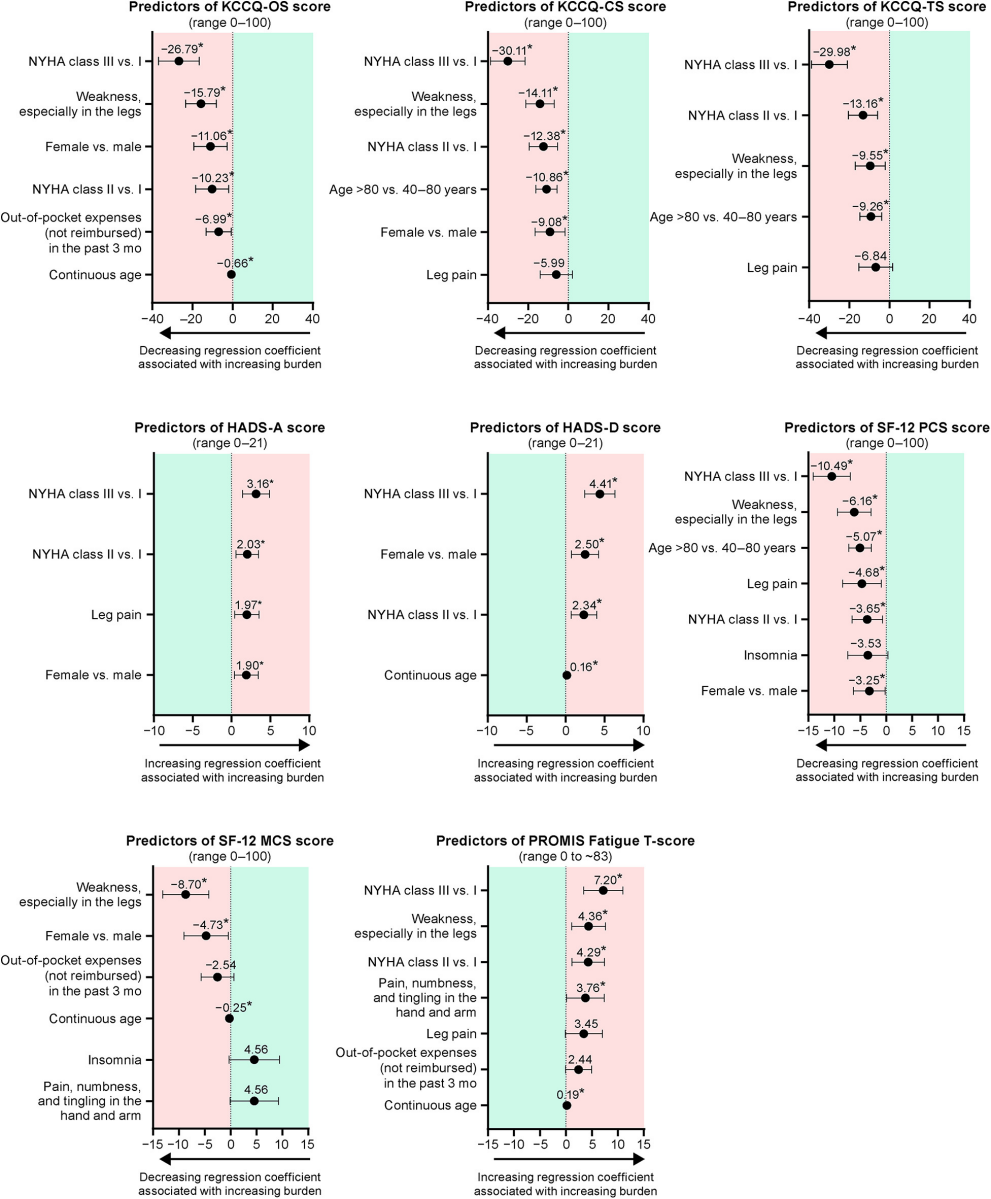
Optimized models for the predictors of patient burden. Each figure shows the regression coefficient with 95% confidence interval. *P < 0.05. CS, clinical summary; HADS-A, Hospital and Depression Scale anxiety; HADS-D, Hospital Anxiety and Depression Scale depression; KCCQ, Kansas City Cardiomyopathy Questionnaire; MCS, mental component summary; NYHA, New York Heart Association; OS, overall summary; PCS, physical component summary; PROMIS, Patient-Reported Outcomes Measurement Information System; SF-12; 12-item Short Form Health Survey; TS, total symptoms.

Findings in the optimized models reflected those from the univariate and full multivariate models that showed higher NYHA functional class, having the symptom “weakness, especially in the legs,” female sex, and older age to be frequently among the best independent predictors of a higher patient burden in several measures (Figure 2; Supplementary Table S3)*.* Having an NYHA class of III (vs. I) was associated with the largest changes in burden, and an NYHA class of III (vs. I) and II (vs. I) were significant predictors of higher burden in all models except SF-12 MCS.

Among current symptoms, “weakness, especially in the legs” was associated with the largest change in burden in all models except HADS. It was a statistically significant predictor of higher burden in the optimized models of KCCQ-OS, -CS, and -TS; SF-12 PCS and MCS; and PROMIS Fatigue scores. The symptom “leg pain” was included in the optimized models for KCCQ -CS and -TS, HADS-A, SF-12 PCS, and PROMIS Fatigue scores, but was only a statistically significant predictor of higher burden in the HADS-A and SF-12 PCS models. In the model for PROMIS Fatigue score, “pain, numbness, and tingling in the hand or arm” was also included as a statistically significant predictor of higher burden. Insomnia was included in the optimized models of SF-12 PCS and MCS, in both cases as a non-significant predictor.

Female sex was a significant predictor of higher burden in optimized models for KCCQ-OS and -CS, HADS-A and -D, and SF-12 PCS and MCS scores. Age (either continuous age or being >80 vs. 40–80 years old) was included in all optimized models except HADS-A score, with older age as a significant predictor of higher burden as assessed by KCCQ-OS, -CS, and -TS; HADS-D; SF-12 PCS and MCS; and PROMIS Fatigue scores. Out-of-pocket expenses were included in the optimized models for KCCQ-OS, SF-12 MCS, and PROMIS Fatigue scores, but was only statistically significant predictor of higher burden in the KCCQ-OS model.

### 3.3 Predictors of burden in caregivers

In the unadjusted univariate models, the patient's KCCQ-OS, HADS-A, and HADS-D scores were significant predictors of the caregiver's continuous ZBI score (Supplementary Table S4) and categorical ZBI classification (Supplementary Table S5). The patient's NYHA class (III vs. I) was also a significant predictor of the caregiver's categorical ZBI classification.

In the full multivariate models, which included all predictors, the patient's KCCQ-OS score was the only significant predictor of the caregiver's continuous ZBI score and categorical ZBI classification (Supplementary Table S6). The regression coefficient for continuous ZBI score was −0.28 [95% confidence interval (CI), −0.41, −0.15; *P* < 0.0001]. The odds ratio for a caregiver to have a ZBI score in the 0–20 range vs. the 21–60 range was 0.96 (95% CI, 0.93, 0.99; *P* = 0.0023).

**Figure 3:**
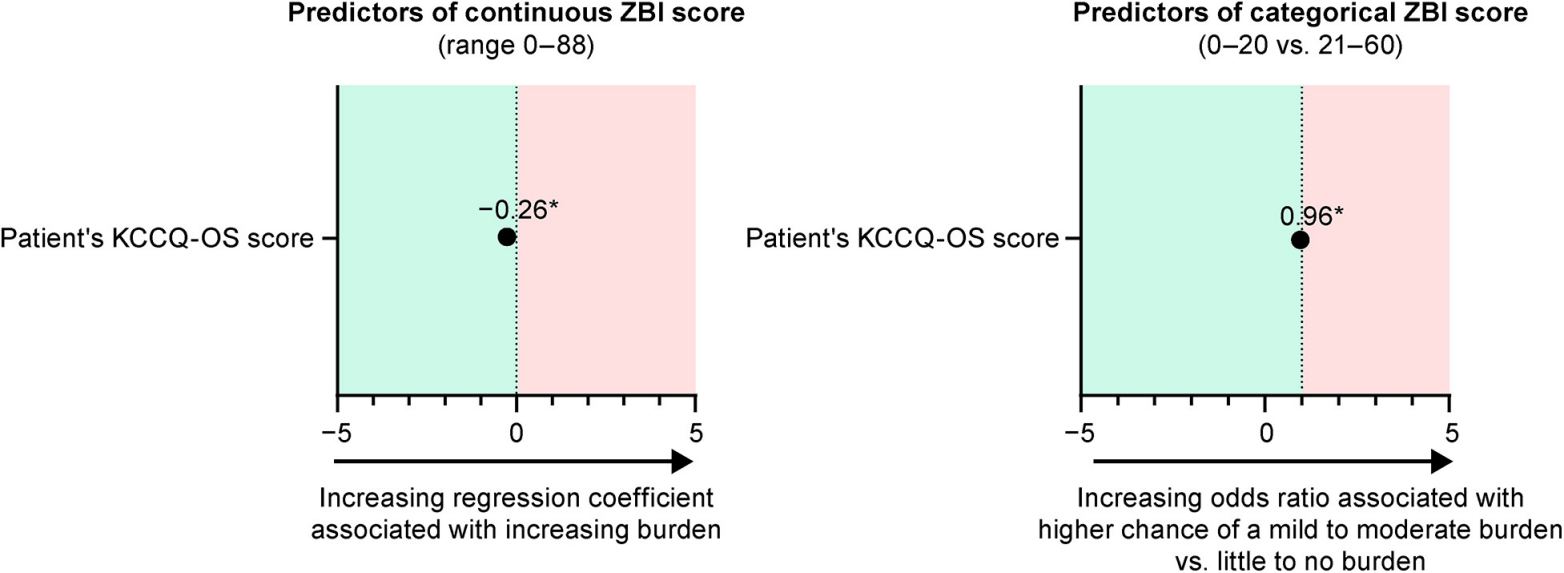
Optimized models for the predictors of caregiver burden. *P < 0.0001. KCCQ, Kansas City Cardiomyopathy Questionnaire; OS, overall summary; ZBI, Zarit Burden Interview.

In the optimized models for caregiver's ZBI score, the patient's KCCQ-OS score was the only remaining predictor (Figure 3). In the model of continuous ZBI score, the regression coefficient was −0.26 (95% CI, −0.33, −0.20; *P* < 0.0001). In the categorical model, the odds ratio for a patient to have no burden (ZBI score, 0–20) vs. a mild to moderate burden (score 21–60) was 0.96 (95% CI, 0.94, 0.97; *P* < 0.0001).

A sensitivity analysis evaluated a range of patient- and caregiver-reported survey items as moderators of the relationship between the patient's KCCQ-OS score and the caregiver's continuous ZBI score. Only a small interaction between the caregiver's SF-12 PCS score and the patient's KCCQ-OS score was statistically significant (regression coefficient, −0.01; *P* = 0.02; Supplementary Table S7). Due to the small regression coefficient, this was not considered a clinically relevant interaction. The relationship between the patient's KCCQ-OS and the caregiver's ZBI was numerically and statistically altered by the addition of caregiver's SF-12 PCS and the interaction to the model, as indicated by a regression coefficient of KCCQ-OS change from −0.27 to 0.14 and significance change from *P* < 0.0001 to *P* = 0.44, but caregiver's SF-12 PCS was not considered to cause a clinically relevant moderator effect.

## 4 Discussion

This study utilized data from a recent international, multicenter, real-world survey to identify predictors of burden in patients with untreated ATTR-CM and their primary, unpaid caregivers (16). Among patients, physical symptoms related to their NYHA functional class and the symptom of “weakness, especially in the legs” were the strongest independent predictors of higher burden, as assessed by several measures. Older age and female sex were also predictors of higher burden in several models. Among caregivers, the patient's KCCQ-OS score (indicating the combined effect of the disease on symptoms, physical limitations, social limitations, and quality of life) was the strongest predictor of burden, as measured in the ZBI score. This relationship was not significantly altered by any single potential patient or caregiver moderator, demonstrating the consistency of this relationship across patients with different disease presentations and in different settings. These findings suggest that symptom control is an important and potentially modifiable determinant of burden in both patients with ATTR-CM and their caregivers. Proactive assessment of and support for bothersome ATTR-CM symptoms could be valuable in clinical practice.

### 4.1 Predictors of patient burden

Each patient outcome measure used in this study describes a slightly different expression of burden, ranging from solely physical to entirely mental burdens. Each has a different scale and magnitude of clinically relevant change. Though not well defined for all scales, or validated in this patient population, clinically relevant changes in KCCQ and SF-12 subscales are proposed to be >5 and ≥3 points, respectively (17, 29–34). For the HADS subscales, a score ≥8 is considered to indicate clinically relevant anxiety or depression (18, 35). Here, a change of a few points may well be relevant, depending on the patient's mental health background. The PROMIS Fatigue scale has a final T score that ranges from 0 to ∼83 (depending on the survey version and conversion algorithm used), with a standardized population mean of 50 and standard deviation of 10 (23, 24). Changes of ≥10 points represent a higher fatigue burden for patients.

We found that having a higher NYHA functional class was a significant independent predictor of higher burden in all optimized models except for SF-12 MCS score, suggesting that it impacts a range of different expressions of burden. Being NYHA class III (vs. I) was associated with the largest regression coefficients of all predictors, ranging from 27 to 30 points in KCCQ, 3–4 in HADS, 10 in SF-12 PCS, and ∼7 in the PROMIS Fatigue scales. The magnitude of these coefficients indicates that being NYHA class III is associated with a considerably larger burden than being class I, and the nature of this burden is broad, including both physical and mental components. An NYHA class of II (vs. I) was also a significant predictor of higher burden in all scales except SF-12 MCS, indicating that a single step increase in NYHA functional classification would have a clinically relevant impact on a patient's disease burden. The coefficients related to an increase from class I to II are often approximately half (range, 35%–64%) that seen from class I to III, further suggesting a proportional relationship between burden and NYHA functional class. This relationship is not unexpected, considering that NYHA class describes both the symptoms and the physical limitations they cause, and is known to be related to KCCQ (17); however, findings from this study confirm that changes in NYHA class are robustly related to multiple dimensions of burden in patients with ATTR-CM.

In addition to heart failure symptoms, other symptoms were also found to be important predictors of patient burden. The symptom of “weakness, especially in the legs” was a significant predictor of higher burden in all optimized models except for the HADS subscales. Regression coefficients indicated this symptom was associated with a 10- to 16-point change in KCCQ subscales, a 9-point change in SF-12 MCS, a 6-point change in SF-12 PCS, and a 4-point change in PROMIS Fatigue T score. Because “weakness, especially in the legs” was the item associated with the largest coefficient in the optimized SF-12 MCS model, and the magnitudes of change seen in KCCQ and SF-12 scales were clinically relevant, it suggests that the HADS poorly captures the mental burden caused by this symptom. Other symptoms showed less consistent associations with patient burden, suggesting that mobility is an important determinant of patient burden. This is supported by literature from other disease areas, also showing that loss of mobility impacts both the mental health and functional abilities of patients (36–40). Several studies suggest that a focus on supporting mobility in patients who are aging or have chronic conditions may help support better outcomes (38, 41, 42).

Female sex and older age also emerged as common independent predictors of higher patient burden. Female sex was associated with a clinically relevant increase in KCCQ-OS and -CS and SF-12 PCS and MCS scores. In the HADS, female sex was associated with a 2- to 3-point increase, which may be relevant, depending on the patient's mental health background. A more substantial impact of heart failure on quality of life in females vs. males has been previously demonstrated, though the reasons behind these sex differences are not known (43). Older age may have disease-independent links with burden, e.g., through the occurrence of comorbidities, increased fragility, and lower resilience; therefore, these should be interpreted with caution. KCCQ scores have previously been shown to decline slightly over a lifetime and become more variable, particularly for symptom and physical limitation dimensions (44, 45). A large study of patients with heart failure found a 7-point decrease in KCCQ-OS across age strata ranging from 32 to ≥80 years (44, 45). Although the SF-12 MCS has been reported to improve ∼2 points over a lifetime, the median PCS is ∼17 points lower in people aged ≥75 years compared with those aged 18–34 years (20). The HADS is reported to be valid in older patients (18).

To our knowledge, this is the first comprehensive statistical assessment of the predictors of burden in patients with ATTR-CM, though a few previous studies have demonstrated the association between NYHA functional class, KCCQ, SF-12 PCS, or EQ-5D-5L scores in similar or other populations (14, 17, 29, 46–48). In the primary survey, from which these *post hoc* study data are derived, patients who were NYHA class III reported numerically higher burden levels than patients who were NYHA class I/II (16). The consistency and magnitude of the predictive power of NYHA class on patient burden is confirmed in this analysis. Although the presence of physical symptoms provides logical causation for higher burden levels, the particular strength of association between “weakness, especially in the legs” and patient burden is novel. In the primary survey, 62% of patients were unable to walk normally, and a previous qualitative study found that intolerance to activity, inability to exercise, insomnia, and fatigue were the most challenging symptoms of ATTR amyloidosis for patients (10, 16). Our findings confirm that ability to complete physical tasks is an important and potentially modifiable contributor to patient burden.

### 4.2 Predictors of caregiver burden

The predictors of burden in caregivers to patients with ATTR-CM have not been previously evaluated with similar analytical detail. In the full and optimized models, the patient's KCCQ-OS score was the only predictor significantly associated with caregiver burden. In the optimized model for continuous ZBI score, a 1-point decrease in patient's KCCQ-OS score (indicating worse health status) was associated with a 0.26-point increase in caregiver's ZBI score (indicating higher burden). We would therefore expect a 5-point change in the patient's KCCQ-OS (which is often considered to be the minimally clinically important difference) to be associated with a 1.3-point change in the caregiver's ZBI score (30, 31). Clinically relevant change in ZBI scores in caregivers have not been defined, though the groupings increase in units of 20 (i.e., little or no burden is 0–20, mild to moderate burden is 21–40, moderate to severe burden is 41–60, and severe burden is 61–88). Therefore, a change of 1 unit is unlikely to be clinically relevant to caregivers, requiring a much larger change in KCCQ-OS to experience a notable change in burden. As mental health has previously been shown to modulate the perceived severity of disease in patients with ATTR-CM (28), we additionally evaluated several patient- and caregiver-reported items as potential moderators of the KCCQ/ZBI relationship, finding none to be clinically relevant. Overall findings from the models suggest that changes in patient burden are not as strongly correlated with changes in caregiver burden as may be imagined at this stage in their disease journey, but that reducing the patient's perceived disease severity is by far the best way to reduce caregiver burden.

### 4.3 Study limitations

These analyses used data from a recently completed international survey (16). As numerous factors modify self-reported burden in patients and caregivers, these results should be interpreted within the populations analyzed, namely patients with a reasonably new diagnosis of untreated ATTR-CM, mostly of the wild-type form, and their primary unpaid caregivers. Findings should not be extrapolated to patients with other forms of amyloidosis or who are receiving disease-modifying treatment. Most patient and caregiver pairs resided in Southern Europe, and there may be potential differences in burden predictors across geographies that could not be fully evaluated using these survey data. Further, NYHA functional classification is related to physical capacity but is not assessed with objective measures, such as by cardiopulmonary exercise testing or the 6-minute walking test. The predictors evaluated in this model were only few of those available from the initial study, and were selected based on author review of the complete survey data and their clinical experience with patients with ATTR-CM. There may be unevaluated combinations of items that are also predictive of burden and these analyses were not exhaustive. Model optimization was conducted using the available survey data, which was incomplete for some survey items. Larger datasets could increase the predictive power of the models. Lastly, no caregivers reported a severe burden in the original study, further analyses that include patients and caregivers with higher burdens may help to identify additional predictive items. This may also have limited the statistical power to identify potential moderators on the relationship identified between the patient's KCCQ-OS score and the caregiver's ZBI score. The relationship between patient and caregiver burden deserves further study.

### 4.4 Conclusions

In this study of patients with ATTR-CM who were not receiving disease-modifying therapy, we found that heart failure symptoms, “weakness, especially in the legs,” older age, and female sex were independent predictors of higher disease burden in several measures. We found burden in their caregivers was best predicted by the patient's KCCQ-OS score, and this relationship was not moderated in a clinically significant way by any other patient- and caregiver-reported demographic or clinical characteristic. Though likely requiring a large change to be meaningful, a poorer health status in the patient was consistently associated with a higher burden to the caregiver. Proactively assessing the physical symptoms of ATTR-CM and implementing strategies to reduce their impact may help to reduce burden experienced by patients and their caregivers.

## Data Availability

Upon request, and subject to review, Pfizer will provide the data that support the findings of this study. Subject to certain criteria, conditions, and exceptions, Pfizer may also provide access to the related individual de-identified participant data. See https://www.pfizer.com/science/clinical-trials/trial-data-and-results for more information.
