# Supplementary material for: Predictors of disease burden in patients with untreated transthyretin amyloid cardiomyopathy and their caregivers: a post hoc analysis of an international survey
Source: Front Cardiovasc Med. 2025 Jun 9;12:1595797. doi: 10.3389/fcvm.2025.1595797 (PMC12183217; doi:10.3389/fcvm.2025.1595797)
Supplement: Supplementary file 1 [file Datasheet1.docx]

**Supplementary Material**

**Predictors of disease burden in patients with untreated transthyretin amyloid cardiomyopathy and their caregivers: a *post hoc* analysis of an international survey**

**Francesco Cappelli^1^*, Lucia Ponti^2^, Kristen Hsu^3^, Thibaud Damy^4^, Eduardo Villacorta^5^, Nicolas Verheyen^6^, Denis Keohane^7^, Ronnie Wang^8^, Monica Ines^9^, Nisith Kumar^7^ and Carmen Munteanu^7^, on behalf of the Burden of Disease Study Investigators**

^1^Tuscan Regional Amyloidosis Referral Centre, Careggi University Hospital, Florence, Italy, ^2^University of Urbino, Urbino, Italy, ^3^Amyloidosis Research Consortium, Newton, Massachusetts, United States, ^4^Henri Mondor Hospital, Paris, France, ^5^Complejo Asistencial Universitario de Salamanca, Salamanca, Spain, ^6^Medical University of Graz, Graz, Austria, ^7^Pfizer Inc, New York, New York, United States, ^8^Pfizer Inc, Groton, Connecticut, United States, ^9^Pfizer Inc, Porto Salvo, Portugal

***Correspondence:** Dr Francesco Cappelli, Tuscan Regional Amyloidosis Referral Centre, Careggi University Hospital, Florence, Italy; cappellif@aou-careggi.toscana.it

**SUPPLEMENTARY TABLE S1. Univariate analyses of the predictors of patient burden.**

|  | **Mean for “yes”** | **Mean for “no”** | **Pooled difference (SD)** | ***P* value from  *t* test** | **Pearson correlation coefficient** | ***P* from correlation coefficient** |
| --- | --- | --- | --- | --- | --- | --- |
| **KCCQ-OS score, *n* = 183** | | | | | | |
| Age >80 (yes) vs. 40–80 (no) yrs | 58.6,  *n* = 92 | 69.0,  *n* = 91 | −10.4 (24.0) | 0.004 | −0.212 | 0.004 |
| Female (yes) vs. male (no) | 50.7,  *n* = 25 | 65.8,  *n* = 158 | −15.1 (24.0) | 0.004 | −0.212 | 0.004 |
| NYHA class I/II (yes) vs. III (no) | 68.6,  *n* = 139 | 43.4,  *n* = 37 | 25.3  (22.3) | <0.001 | 0.421 | <0.001 |
| Symptoms (yes vs. no) | | | | | | |
| Weakness, especially in legs | 43.8,  *n* = 38 | 69.0,  *n* = 145 | −25.2 (22.3) | <0.001 | −0.418 | <0.001 |
| Leg pain | 48.8,  *n* = 26 | 66.2,  *n* = 157 | −17.4 (23.8) | <0.001 | −0.249 | <0.001 |
| Pain, numbness, and tingling in the hand and arm | 57.9,  *n* = 23 | 64.6,  *n* = 160 | −6.7 (24.4) | 0.222 | −0.091 | 0.222 |
| Erectile dysfunction | 58.7,  *n* = 26 | 64.6,  *n* = 157 | −5.9 (24.4) | 0.252 | −0.085 | 0.252 |
| Insomnia | 52.4,  *n* = 26 | 65.7,  *n* = 157 | −13.2 (24.1) | 0.010 | −0.189 | 0.010 |
| Dizziness (orthostatic hypotension or syncope) | 55.4,  *n* = 32 | 65.5,  *n* = 151 | −10.1 (24.2) | 0.034 | −0.157 | 0.034 |
| Gastrointestinal or urinary problems | 54.1,  *n* = 32 | 65.8,  *n* = 151 | −11.8 (24.1) | 0.013 | −0.183 | 0.013 |
| Out-of-pocket expenses (not reimbursed) in the last 3 months, yes vs. no | 55.0,  *n* = 70 | 69.0,  *n* = 105 | −14.0 (23.9) | <0.001 | −0.277 | <0.001 |
| **KCCQ-CS score, *n* = 202** | | | | | | |
| Age >80 (yes) vs. 40–80 (no) yrs | 61.2,  *n* = 104 | 73.2,  *n* = 98 | −12.0 (23.2) | <0.001 | −0.251 | <0.001 |
| Female (yes) vs. male (no) | 55.8,  *n* = 27 | 68.7,  *n* = 175 | −12.9 (23.6) | 0.009 | −0.184 | 0.009 |
| NYHA class I/II (yes) vs. III (no) | 72.7,  *n* = 151 | 47.1,  *n* = 42 | 25.7  (21.3) | <0.001 | 0.448 | <0.001 |
| Symptoms (yes vs. no) | | | | | | |
| Weakness, especially in legs | 49.3,  *n* = 40 | 71.4,  *n* = 162 | −22.1 (22.3) | <0.001 | −0.368 | <0.001 |
| Leg pain | 52.4,  *n* = 27 | 69.3,  *n* = 175 | −16.8 (23.3) | <0.001 | −0.240 | <0.001 |
| Pain, numbness, and tingling in the hand and arm | 60.2,  *n* = 25 | 68.0,  *n* = 177 | −7.8 (23.9) | 0.129 | −0.107 | 0.129 |
| Erectile dysfunction | 61.3,  *n* = 29 | 68.0,  *n* = 173 | −6.7 (23.9) | 0.162 | −0.099 | 0.162 |
| Insomnia | 54.6,  *n* = 28 | 69.0,  *n* = 174 | −14.4 (23.5) | 0.003 | −0.208 | 0.003 |
| Dizziness (orthostatic hypotension or syncope) | 58.4,  *n* = 33 | 68.7,  *n* = 169 | −10.3 (23.7) | 0.024 | −0.159 | 0.024 |
| Gastrointestinal or urinary problems | 56.2,  *n* = 34 | 69.2,  *n* = 168 | −13.0 (23.5) | 0.004 | −0.204 | 0.004 |
| Out-of-pocket expenses (not reimbursed) in the last 3 months, yes vs. no | 60.6,  *n* = 76 | 70.6,  *n* = 117 | −10.0 (23.8) | 0.005 | −0.202 | 0.005 |
| **KCCQ-TS score, *n* = 208** | | | | | | |
| Age >80 (yes) vs. 40–80 (no) yrs | 65.6,  *n* = 108 | 76.0,  *n* = 100 | −10.4 (23.4) | 0.001 | −0.218 | 0.002 |
| Female (yes) vs. male (no) | 68.6,  *n* = 29 | 70.9,  *n* = 179 | −2.3 (24.0) | 0.634 | −0.033 | 0.634 |
| NYHA class I/II (yes) vs. III (no) | 76.1,  *n* = 156 | 52.6,  *n* = 43 | 23.5  (21.4) | <0.001 | 0.413 | <0.001 |
| Symptoms (yes vs. no) | | | | | | |
| Weakness, especially in legs | 56.0,  *n* = 41 | 74.2,  *n* = 167 | −18.2 (22.9) | <0.001 | −0.303 | <0.001 |
| Leg pain | 56.9,  *n* = 28 | 72.7,  *n* = 180 | −15.9 (23.4) | <0.001 | −0.227 | <0.001 |
| Pain, numbness, and tingling in the hand and arm | 65.3,  *n* = 25 | 71.3,  *n* = 183 | −6.1 (23.9) | 0.234 | −0.083 | 0.234 |
| Erectile dysfunction | 66.4,  *n* = 30 | 71.3,  *n* = 178 | −4.9 (23.9) | 0.302 | −0.072 | 0.302 |
| Insomnia | 61.2,  *n* = 29 | 72.1,  *n* = 179 | −11.0 (23.7) | 0.022 | −0.159 | 0.022 |
| Dizziness (orthostatic hypotension or syncope) | 66.1,  *n* = 33 | 71.5,  *n* = 175 | −5.4 (23.9) | 0.239 | −0.082 | 0.239 |
| Gastrointestinal or urinary problems | 62.8,  *n* = 36 | 72.2,  *n* = 172 | −9.5 (23.7) | 0.031 | −0.150 | 0.031 |
| Out-of-pocket expenses (not reimbursed) in the last 3 months, yes vs. no | 68.5,  *n* = 78 | 71.3,  *n* = 121 | −2.7 (24.3) | 0.437 | −0.055 | 0.437 |
| **HADS-A score, *n* = 205** | | | | | | |
| Age >80 (yes) vs. 40–80 (no) yrs | 5.7,  *n* = 106 | 5.4,  *n* = 99 | 0.3 (4.1) | 0.596 | 0.037 | 0.596 |
| Female (yes) vs. male (no) | 7.2,  *n* = 29 | 5.3,  *n* = 176 | 1.9 (4.0) | 0.019 | 0.164 | 0.019 |
| NYHA class I/II (yes) vs. III (no) | 5.1,  *n* = 153 | 7.1,  *n* = 43 | −2.0 (4.0) | 0.004 | −0.205 | 0.004 |
| Symptoms (yes vs. no) | | | | | | |
| Weakness, especially in legs | 6.7,  *n* = 41 | 5.3,  *n* = 164 | 1.5 (4.1) | 0.040 | 0.144 | 0.040 |
| Leg pain | 7.5,  *n* = 28 | 5.3,  *n* = 177 | 2.2 (4.0) | 0.007 | 0.189 | 0.007 |
| Pain, numbness, and tingling in the hand and arm | 5.5,  *n* = 25 | 5.6,  *n* = 180 | −0.0 (4.1) | 0.957 | −0.004 | 0.957 |
| Erectile dysfunction | 5.6,  *n* = 30 | 5.5,  *n* = 175 | 0.1 (4.1) | 0.917 | 0.007 | 0.917 |
| Insomnia | 6.3,  *n* = 29 | 5.4,  *n* = 176 | 0.8 (4.1) | 0.310 | 0.071 | 0.310 |
| Dizziness (orthostatic hypotension or syncope) | 5.8,  *n* = 33 | 5.5,  *n* = 172 | 0.2 (4.1) | 0.764 | 0.021 | 0.764 |
| Gastrointestinal or urinary problems | 5.9,  *n* = 36 | 5.5,  *n* = 169 | 0.4 (4.1) | 0.629 | 0.034 | 0.629 |
| Out-of-pocket expenses (not reimbursed) in the last 3 months, yes vs. no | 6.3,  *n* = 77 | 5.2,  *n* = 119 | 1.1 (4.1) | 0.066 | 0.132 | 0.066 |
| **HADS-D score, *n* = 204** | | | | | | |
| Age >80 (yes) vs. 40–80 (no) yrs | 7.9,  *n* = 105 | 5.7,  *n* = 99 | 2.2 (4.8) | 0.001 | 0.222 | 0.001 |
| Female (yes) vs. male (no) | 9.3,  *n* = 28 | 6.4,  *n* = 176 | 2.9 (4.8) | 0.004 | 0.202 | 0.004 |
| NYHA class I/II (yes) vs. III (no) | 6.2,  *n* = 152 | 9.0,  *n* = 43 | −2.9 (4.7) | <0.001 | −0.248 | <0.001 |
| Symptoms (yes vs. no) | | | | | | |
| Weakness, especially in legs | 8.5,  *n* = 41 | 6.4,  *n* = 163 | 2.1 (4.8) | 0.013 | 0.174 | 0.013 |
| Leg pain | 8.1,  *n* = 27 | 6.6,  *n* = 177 | 1.5 (4.9) | 0.140 | 0.104 | 0.140 |
| Pain, numbness, and tingling in the hand and arm | 7.3,  *n* = 25 | 6.7,  *n* = 179 | 0.6 (4.9) | 0.585 | 0.038 | 0.585 |
| Erectile dysfunction | 6.5,  *n* = 30 | 6.9,  *n* = 174 | −0.3 (4.9) | 0.730 | −0.024 | 0.730 |
| Insomnia | 7.5,  *n* = 28 | 6.7,  *n* = 176 | 0.7 (4.9) | 0.453 | 0.053 | 0.453 |
| Dizziness (orthostatic hypotension or syncope) | 7.6,  *n* = 33 | 6.7,  *n* = 171 | 0.9 (4.9) | 0.313 | 0.071 | 0.313 |
| Gastrointestinal or urinary problems | 8.3,  *n* = 35 | 6.5,  *n* = 169 | 1.8 (4.8) | 0.051 | 0.137 | 0.051 |
| Out-of-pocket expenses (not reimbursed) in the last 3 months, yes vs. no | 8.1,  *n* = 76 | 6.2,  *n* = 119 | 1.8 (4.9) | 0.010 | 0.183 | 0.010 |
| **SF-12 PCS score, *n* = 206** | | | | | | |
| Age >80 (yes) vs. 40–80 (no) yrs | 33.2,  *n* = 107 | 38.6,  *n* = 99 | −5.4 (9.4) | <0.001 | −0.277 | <0.001 |
| Female (yes) vs. male (no) | 32.1,  *n* = 29 | 36.3,  *n* = 177 | −4.2 (9.7) | 0.029 | −0.152 | 0.029 |
| NYHA class I/II (yes) vs. III (no) | 37.9,  *n* = 154 | 27.7,  *n* = 43 | 10.1 (8.8) | <0.001 | 0.430 | <0.001 |
| Symptoms (yes vs. no) | | | | | | |
| Weakness, especially in legs | 28.8,  *n* = 41 | 37.5,  *n* = 165 | −8.7 (9.1) | <0.001 | −0.358 | <0.001 |
| Leg pain | 29.0,  *n* = 28 | 36.8,  *n* = 178 | −7.8 (9.4) | <0.001 | −0.276 | <0.001 |
| Pain, numbness, and tingling in the hand and arm | 33.0,  *n* = 25 | 36.1,  *n* = 181 | −3.2 (9.7) | 0.128 | −0.106 | 0.128 |
| Erectile dysfunction | 32.3,  *n* = 30 | 36.3,  *n* = 176 | −4.1 (9.7) | 0.033 | −0.149 | 0.033 |
| Insomnia | 32.2,  *n* = 29 | 36.3,  *n* = 177 | −4.2 (9.7) | 0.032 | −0.149 | 0.032 |
| Dizziness (orthostatic hypotension or syncope) | 32.9,  *n* = 33 | 36.3,  *n* = 173 | −3.4 (9.7) | 0.063 | −0.130 | 0.063 |
| Gastrointestinal or urinary problems | 31.9,  *n* = 36 | 36.6,  *n* = 170 | −4.7 (9.6) | 0.008 | −0.183 | 0.008 |
| Out-of-pocket expenses (not reimbursed) in the last 3 months, yes vs. no | 33.5,  *n* = 77 | 37.0,  *n* = 120 | −3.4 (9.7) | 0.016 | −0.171 | 0.016 |
| **SF-12 MCS score, *n* = 206** | | | | | | |
| Age >80 (yes) vs. 40–80 (no) yrs | 45.1,  *n* = 107 | 47.1,  *n* = 99 | −2.1 (11.4) | 0.195 | −0.091 | 0.195 |
| Female (yes) vs. male (no) | 42.1,  *n* = 29 | 46.7,  *n* = 177 | −4.6 (11.3) | 0.042 | −0.142 | 0.042 |
| NYHA class I/II (yes) vs. III (no) | 46.9,  *n* = 154 | 42.4,  *n* = 43 | 4.5  (11.3) | 0.023 | 0.162 | 0.023 |
| Symptoms (yes vs. no) | | | | | | |
| Weakness, especially in legs | 41.5,  *n* = 41 | 47.2,  *n* = 165 | −5.7 (11.2) | 0.004 | −0.200 | 0.004 |
| Leg pain | 44.4,  *n* = 28 | 46.3,  *n* = 178 | −2.0 (11.4) | 0.396 | −0.059 | 0.396 |
| Pain, numbness, and tingling in the hand and arm | 48.6,  *n* = 25 | 45.7,  *n* = 181 | 2.8  (11.4) | 0.244 | 0.082 | 0.244 |
| Erectile dysfunction | 46.9,  *n* = 30 | 45.9,  *n* = 176 | 1.0  (11.4) | 0.651 | 0.032 | 0.651 |
| Insomnia | 45.3,  *n* = 29 | 46.2,  *n* = 177 | −0.9 (11.4) | 0.692 | −0.028 | 0.692 |
| Dizziness (orthostatic hypotension or syncope) | 44.2,  *n* = 33 | 46.4,  *n* = 173 | −2.2 (11.4) | 0.300 | −0.073 | 0.300 |
| Gastrointestinal or urinary problems | 44.1,  *n* = 36 | 46.5,  *n* = 170 | −2.4 (11.4) | 0.252 | −0.080 | 0.252 |
| Out-of-pocket expenses (not reimbursed) in the last 3 months, yes vs. no | 43.5,  *n* = 77 | 47.6,  *n* = 120 | −4.1 (11.3) | 0.014 | −0.175 | 0.014 |
| **PROMIS Fatigue score, *n* = 203** | | | | | | |
| Age >80 (yes) vs. 40–80 (no) yrs | 52.6,  *n* = 103 | 50.1,  *n* = 100 | 2.5 (9.4) | 0.062 | 0.131 | 0.062 |
| Female (yes) vs. male (no) | 53.5,  *n* = 29 | 51.0,  *n* = 174 | 2.4 (9.4) | 0.198 | 0.091 | 0.198 |
| NYHA class I/II (yes) vs. III (no) | 50.2,  *n* = 152 | 56.6,  *n* = 43 | −6.4 (9.0) | <0.001 | −0.284 | <0.001 |
| Symptoms (yes vs. no) | | | | | | |
| Weakness, especially in legs | 58.3,  *n* = 41 | 49.6,  *n* = 162 | 8.7 (8.8) | <0.001 | 0.372 | <0.001 |
| Leg pain | 58.2,  *n* = 28 | 50.3,  *n* = 175 | 7.9 (9.0) | <0.001 | 0.290 | <0.001 |
| Pain, numbness, and tingling in the hand and arm | 56.3,  *n* = 25 | 50.7,  *n* = 178 | 5.6 (9.3) | 0.005 | 0.197 | 0.005 |
| Erectile dysfunction | 54.0,  *n* = 30 | 50.9,  *n* = 173 | 3.1 (9.4) | 0.097 | 0.117 | 0.097 |
| Insomnia | 55.5,  *n* = 29 | 50.7,  *n* = 174 | 4.8 (9.3) | 0.010 | 0.180 | 0.010 |
| Dizziness (orthostatic hypotension or syncope) | 53.6,  *n* = 33 | 51.0,  *n* = 170 | 2.6 (9.4) | 0.147 | 0.102 | 0.147 |
| Gastrointestinal or urinary problems | 54.8,  *n* = 36 | 50.6,  *n* = 167 | 4.1 (9.3) | 0.016 | 0.169 | 0.016 |
| Out-of-pocket expenses (not reimbursed) in the last 3 months, yes vs. no | 54.2,  *n* = 75 | 49.7,  *n* = 119 | 4.5 (9.3) | 0.001 | 0.23 | 0.001 |
| CS, Clinical Summary; HADS-A, Hospital and Depression Scale anxiety; HADS-D, Hospital Anxiety and Depression Scale depression; KCCQ, Kansas City Cardiomyopathy Questionnaire; MCS, Mental Component Summary; NYHA, New York Heart Association; OS, Overall Summary; PCS, Physical Component Summary; PROMIS, Patient-Reported Outcomes Measurement Information System; SD, standard deviation; SF-12, 12-item Short Form Health Survey; TS, Total Symptoms. | | | | | | |

**SUPPLEMENTARY TABLE S2. Multivariate models of the predictors of patient burden.**

|  | **KCCQ-OS**  ***n* = 168** | | **KCCQ-CS**  ***n* = 184** | | **KCCQ-TS**  ***n* = 190** | | **HADS-A**  ***n* = 187** | | **HADS-D**  ***n* = 186** | | **SF-12 PCS**  ***n* = 188** | | **SF-12 MCS**  ***n* = 188** | | **PROMIS Fatigue**  ***n* = 186** | |
| --- | --- | --- | --- | --- | --- | --- | --- | --- | --- | --- | --- | --- | --- | --- | --- | --- |
|  | **RC** | ***P* value** | **RC** | ***P* value** | **RC** | ***P* value** | **RC** | ***P* value** | **RC** | ***P* value** | **RC** | ***P* value** | **RC** | ***P* value** | **RC** | ***P* value** |
| Age, yrs |  |  |  |  |  |  |  |  |  |  |  |  |  |  |  |  |
| Continuous | −0.63 (−1.05,  −0.22) | 0.0030 | −0.73  (−1.11,  −0.34) | 0.0002 | - | - | 0.05  (−0.03, 0.13) | 0.1888 | 0.16  (0.07, 0.25) | 0.0004 | - | - | −0.22  (−0.43, 0.00) | 0.0513 | 0.18 (0.01, 0.35) | 0.0336 |
| >80 vs. 40–80 | - | - | - | - | −8.89  (−14.70, −3.08) | 0.0027 | - | - | - | - | −4.92 (−7.25, −2.59) | <0.0001 | - | - | - | - |
| Sex, female vs. male | −10.60  (−19.15,  −2.05) | 0.0151 | −7.78 (−15.67, 0.12) | 0.0534 | 0.44  (−7.67, 8.54) | 0.9159 | 1.93 (0.33, 3.54) | 0.0184 | 2.20  (0.40, 4.00) | 0.0167 | −3.14 (−6.38, 0.09) | 0.0569 | -4.57 (−8.94,  −0.21) | 0.040 | 1.12  (−2.22, 4.47) | 0.5099 |
| NYHA class |  |  |  |  |  |  |  |  |  |  |  |  |  |  |  |  |
| Class II vs. I | −10.78 (−19.13,  −2.43) | 0.0114 | −10.80 (−18.26, −3.34) | 0.0045 | −13.97  (−21.67, −6.27) | 0.0004 | 1.86 (0.30, 3.42) | 0.0194 | 1.99 (0.25, 3.72) | 0.0245 | −3.64 (−6.72,  −0.55) | 0.0208 | −4.31 (−8.47,  −0.14) | 0.0426 | 4.41 (1.22, 7.60) | 0.0067 |
| Class III vs. I | −26.64 (−36.86,  −16.42) | <0.0001 | −28.63 (−37.65, −19.61) | <0.0001 | −30.53 (−39.92, −21.14) | <0.0001 | 3.04 (1.16, 4.92) | 0.0015 | 3.60 (1.52, 5.68) | 0.0007 | −10.68 (−14.42, −6.93) | <0.0001 | −5.86 (−10.92, −0.80) | 0.0231 | 7.03 (3.15, 10.90) | 0.0004 |
| Symptoms, yes vs. no |  |  |  |  |  |  |  |  |  |  |  |  |  |  |  |  |
| Weakness, especially in legs | −15.69 (−24.85,  −6.53) | 0.0008 | −12.55 (−20.77, −4.33) | 0.0028 | −11.55 (−20.25, −2.85) | 0.0093 | 0.91 (−0.81, 2.63) | 0.3013 | 1.62 (−0.31, 3.54) | 0.0996 | −5.47 (−8.94,  −1.99) | 0.002 | −7.24 (−11.93, −2.56) | 0.0024 | 5.60 (2.02, 9.19) | 0.0022 |
| Leg pain | −9.54 (−20.34, 1.27) | 0.0836 | −7.75 (−17.21, 1.71) | 0.1084 | −10.99 (−21.02, −0.96) | 0.0318 | 2.44 (0.46, 4.43) | 0.0157 | 1.14 (−1.10, 3.37) | 0.3191 | −4.60 (−8.60,  −0.59) | 0.0244 | −1.25 (−6.64, 4.14) | 0.6499 | 4.85 (0.73, 8.98) | 0.0211 |
| Pain, numbness, and tingling in the hand and arm | 3.50 (−6.16, 13.15) | 0.4781 | 0.68 (−8.23, 9.59) | 0.8814 | 0.76  (−8.62, 10.14) | 0.8743 | −0.75 (−2.62, 1.11) | 0.4280 | −0.16 (−2.23, 1.90) | 0.8772 | 0.45 (−3.30, 4.19) | 0.8148 | 5.91 (0.84, 10.97) | 0.0222 | 3.92 (0.05, 7.80) | 0.0473 |
| Erectile dysfunction | 4.97 (−5.13, 15.07) | 0.3350 | 4.09 (−5.01, 13.20) | 0.3783 | 7.78 (−1.85, 17.41) | 0.1132 | −0.29 (−2.20, 1.62) | 0.7688 | −0.99 (−3.13, 1.14) | 0.3606 | −0.28 (−4.12, 3.56) | 0.8861 | 0.70 (−4.49, 5.89) | 0.7921 | −0.96 (−4.93, 3.01) | 0.6341 |
| Insomnia | 4.61 (−6.17, 15.38) | 0.4020 | −0.36 (−9.85, 9.13) | 0.9408 | 2.25 (−7.81, 12.31) | 0.6613 | −1.21 (−3.19, 0.78) | 0.2345 | −1.32 (−3.55, 0.91) | 0.2452 | 3.54 (−0.47, 7.56) | 0.0837 | 5.15 (−0.26, 10.55) | 0.0620 | −2.44 (−6.57, 1.70) | 0.2481 |
| Dizziness (orthostatic hypotension or syncope) | −1.11 (−9.83, 7.61) | 0.8030 | −1.14 (−9.21, 6.93) | 0.7813 | 1.19 (−7.37, 9.75) | 0.7852 | 0.04 (−1.64, 1.72) | 0.9644 | 0.03 (−1.84, 1.90) | 0.9741 | −1.11 (−4.53, 2.30) | 0.5224 | −1.73 (−6.31, 2.84) | 0.4572 | −1.26 (−4.76, 2.23) | 0.4788 |
| Gastrointestinal or urinary problems | −2.69 (−11.32, 5.94) | 0.5409 | −3.40  (−11.42, 4.61) | 0.4054 | −2.63  (−10.89, 5.63) | 0.5319 | −0.79  (−2.43, 0.84) | 0.3415 | 0.60 (−1.25, 2.44) | 0.5278 | 0.53 (−2.77, 3.83) | 0.7532 | −0.39 (−4.84, 4.06) | 0.8631 | 0.52 (−2.89, 3.94) | 0.7635 |
| Out-of-pocket expenses (not reimbursed) in the last 3 months, yes vs. no | −6.05 (−12.36, 0.26) | 0.0601 | −3.81 (−9.58, 1.96) | 0.1953 | 3.65 (−2.36, 9.66) | 0.2335 | 0.31 (−0.89, 1.52) | 0.6083 | 0.99 (−0.35, 2.33) | 0.1460 | −0.76  (−3.17, 1.65) | 0.5384 | −1.51 (−4.76, 1.74) | 0.3638 | 2.23 (−0.28, 4.73) | 0.080 |
| Shows regression coefficient with 95% confidence interval for predictors in the full models.  CS, Clinical Summary; HADS-A, Hospital and Depression Scale anxiety; HADS-D, Hospital Anxiety and Depression Scale depression; KCCQ, Kansas City Cardiomyopathy Questionnaire; MCS, Mental Component Summary; OS, Overall Summary; NYHA, New York Heart Association; PCS, Physical Component Summary; PROMIS, Patient-Reported Outcomes Measurement Information System; RC, regression coefficient; SF-12, 12-item Short Form Health Survey; TS, Total Symptoms. | | | | | | | | | | | | | | | | |

**SUPPLEMENTARY TABLE S3.** **Optimized models of the predictors of patient burden.**

|  | **KCCQ-OS**  ***n* = 168** | | **KCCQ-CS**  ***n* = 184** | | **KCCQ-TS**  ***n* = 190** | | **HADS-A**  ***n* = 187** | | **HADS-D**  ***n* = 186** | | **SF-12 PCS**  ***n* = 188** | | **SF-12 MCS**  ***n* = 188** | | **PROMIS Fatigue**  ***n* = 186** | |
| --- | --- | --- | --- | --- | --- | --- | --- | --- | --- | --- | --- | --- | --- | --- | --- | --- |
|  | **RC** | ***P* value** | **RC** | ***P* value** | **RC** | ***P* value** | **RC** | ***P* value** | **RC** | ***P* value** | **RC** | ***P* value** | **RC** | ***P* value** | **RC** | ***P* value** |
| Age, yrs |  |  |  |  |  |  |  |  |  |  |  |  |  |  |  |  |
| Continuous | −0.66 (−1.08,  −0.25) | 0.0016 | - | - | - | - | - | - | 0.16 (0.07, 0.25) | 0.0003 | - | - | −0.25 (−0.46,  −0.04) | 0.0213 | 0.19 (0.03, 0.35) | 0.0234 |
| >80 vs. 40–80 | - | - | −10.86 (−16.07, −5.65) | <0.0001 | −9.26  (−14.67, −3.85) | 0.0008 | - | - | - | - | −5.07 (−7.25,  −2.89) | <0.0001 | - | - | - | - |
| Sex, female vs. male | −11.06 (−19.36, −2.75) | 0.0091 | −9.08 (−16.57, −1.59) | 0.0174 | - | - | 1.90 (0.37, 3.42) | 0.0147 | 2.50 (0.75, 4.24) | 0.0051 | −3.25 (−6.32,  −0.18) | 0.0380 | −4.73 (−9.00,  −0.45) | 0.0303 | - | - |
| NYHA class |  |  |  |  |  |  |  |  |  |  |  |  |  |  |  |  |
| Class II vs. I | −10.23 (−18.46, −2.00) | 0.0148 | −12.38 (−19.42, −5.34) | 0.0006 | −13.16 (−20.44, −5.88) | 0.0004 | 2.03 (0.58, 3.48) | 0.0061 | 2.34 (0.70, 3.99) | 0.0053 | −3.65 (−6.57,  −0.72) | 0.0145 | - | - | 4.29 (1.13, 7.44) | 0.0078 |
| Class III vs. I | −26.79 (−36.88, −16.71) | <0.0001 | −30.11 (−38.69, −21.53) | <0.0001 | −29.98 (−38.89, −21.06) | <0.0001 | 3.16 (1.43, 4.89) | 0.0003 | 4.41 (2.48, 6.35) | <0.0001 | −10.49 (−14.06, −6.93) | <0.0001 | - | - | 7.20 (3.41, 10.99) | 0.0002 |
| Symptoms, yes vs. no |  |  |  |  |  |  |  |  |  |  |  |  |  |  |  |  |
| Weakness, especially in legs | −15.79 (−23.45, −8.12) | <0.0001 | −14.11 (−21.20, −7.03) | <0.0001 | −9.55 (−17.04, −2.07) | 0.0124 | - | - | - | - | −6.16 (−9.38,  −2.93) | 0.0002 | −8.70 (−13.14, −4.25) | 0.0001 | 4.36 (1.11, 7.61) | 0.0086 |
| Leg pain | - | - | −5.99 (−14.05, 2.06) | 0.1446 | −6.84 (−15.31, 1.63) | 0.1136 | 1.97 (0.41, 3.53) | 0.0135 | - | - | −4.68 (−8.41,  −0.95) | 0.0139 | - | - | 3.45 (-0.13, 7.03) | 0.0588 |
| Pain, numbness, and tingling in the hand and arm | - | - | - | - | - | - | - | - | - | - | - | - | 4.56 (−0.12, 9.24) | 0.0559 | 3.76 (0.13, 7.39) | 0.0424 |
| Erectile dysfunction | - | - | - | - | - | - | - | - | - | - | - | - | - | - | - | - |
| Insomnia | - | - | - | - | - | - | - | - | - | - | −3.53 (−7.42, 0.36) | 0.0752 | 4.56 (−0.31, 9.42) | 0.0663 | - | - |
| Dizziness (orthostatic hypotension or syncope) | - | - | - | - | - | - | - | - | - | - | - | - | - | - | - | - |
| Gastrointestinal or urinary problems | - | - | - | - | - | - | - | - | - | - | - | - | - | - | - | - |
| Out-of-pocket expenses (not reimbursed) in the last 3 months,  yes vs. no | −6.99 (−13.26, −0.72) | 0.0289 | - | - | - | - | - | - | - | - | - | - | −2.54 (−5.70, 0.62) | 0.1152 | 2.44 (−0.05, 4.93) | 0.0543 |
| Shows regression coefficient with 95% confidence interval for all predictors remaining in the optimized models. Predictors without regression coefficients were not included in the optimized models.  CS, Clinical Summary; HADS-A, Hospital and Depression Scale anxiety; HADS-D, Hospital Anxiety and Depression Scale depression; KCCQ, Kansas City Cardiomyopathy Questionnaire; MCS, Mental Component Summary; NYHA, New York Heart Association; OS, Overall Summary; PCS, Physical Component Summary; PROMIS, Patient-Reported Outcomes Measurement Information System; RC, regression coefficient; SF-12, 12-item Short Form Health Survey; TS, Total Symptoms. | | | | | | | | | | | | | | | | |

**SUPPLEMENTARY TABLE S4. Univariate analyses of the predictors of caregiver’s continuous ZBI score.**

|  | **Continuous ZBI score** | | | | |
| --- | --- | --- | --- | --- | --- |
|  | ***n*** | **Mean (SD)** | ***P* from linear regression** | **Pearson correlation coefficient** | ***P* from correlation coefficient** |
| Patient’s NYHA class | | | | | |
| II vs. I | 120 vs. 36 | 15.8 (13.3) vs.  12.6 (11.5) | 0.194 | 0.137 | 0.054 |
| III vs. I | 43 vs. 36 | 18.3 (13.6) vs.  12.6 (11.5) | 0.051 |  |  |
| Spouse of the patient vs. other relationship | 122 vs. 86 | 14.2 (12.8) vs. 16.5 (13.6) | 0.217 | 0.085 | 0.221 |
| Male vs. female | 32 vs. 176 | 12.3 (13.4) vs. 15.7 (13.1) | 0.170 | −0.095 | 0.173 |
| Lives with the patient, no vs. yes | 70 vs. 138 | 16.6 (13.4) vs. 14.4 (13.0) | 0.266 | 0.077 | 0.269 |
| Hours per week spent with the patient | 196 | - | 0.652 | −0.032 | 0.654 |
| Years spent providing care to the patient | 166 | - | 0.980 | −0.002 | 0.980 |
| Patient’s KCCQ-OS score | 183 | - | <0.001 | −0.494 | <0.001 |
| Patient’s HADS-A score | 205 | - | <0.001 | 0.361 | <0.001 |
| Patient’s HADS-D score | 204 | - | <0.001 | 0.342 | <0.001 |
| HADS-A, Hospital and Depression Scale anxiety; HADS-D, Hospital Anxiety and Depression Scale depression; KCCQ, Kansas City Cardiomyopathy Questionnaire; OS, Overall Summary; NYHA, New York Heart Association; SD, standard deviation; ZBI, Zarit Burden Interview. | | | | | |

**SUPPLEMENTARY TABLE S5. Univariate analyses of the predictors of caregiver’s categorical ZBI classification.**

|  | **Categorical ZBI classification** | | | | |
| --- | --- | --- | --- | --- | --- |
|  | ***n*/*N* (%)** | | ***P* from ordinal logistic regression** | **Pearson correlation coefficient** | ***P* from correlation coefficient** |
|  | **0–20** | **21–60** |  |  |  |
| Patient’s NYHA class | | | | | |
| I | 27/36 (75.0) | 9/36 (25.0) | Ref | 0.172 | 0.015 |
| II | 77/120 (64.2) | 43/120 (35.8) | 0.229 |  |  |
| III | 21/43 (48.8) | 22/43 (51.2) | 0.020 |  |  |
| Relationship to patient | | | | | |
| Spouse | 83/122 (68.0) | 39/122 (32.0) | 0.196 | 0.090 | 0.197 |
| Other | 51/86 (59.3) | 35/86 (40.7) | Ref |  |  |
| Sex | | | | | |
| Female | 111/176 (63.1) | 65/176 (36.9) | Ref | −0.066 | 0.341 |
| Male | 23/32 (71.9) | 9/32 (28.1) | 0.341 |  |  |
| Lives with the patient | | | | | |
| Yes | 91/138 (65.9) | 47/138 (34.1) | Ref | 0.045 | 0.523 |
| No | 43/70 (61.4) | 27/70 (38.6) | 0.521 |  |  |
| Hours per week spent with the patient | - | - | 0.933 | 0.006 | 0.933 |
| Years spent providing care to the patient | - | - | 0.825 | –0.017 | 0.826 |
| Patient’s KCCQ-OS score | - | - | <0.001 | –0.456 | <0.001 |
| Patient’s HADS-A score | - | - | <0.001 | 0.330 | <0.001 |
| Patient’s HADS-D score | - | - | <0.001 | 0.295 | <0.001 |
| HADS-A, Hospital and Depression Scale anxiety; HADS-D, Hospital Anxiety and Depression Scale depression; KCCQ, Kansas City Cardiomyopathy Questionnaire; OS, Overall Summary; NYHA, New York Heart Association; ZBI, Zarit Burden Interview. | | | | | |

**SUPPLEMENTARY TABLE S6. Multivariate model of the predictors of caregiver’s ZBI score.**

|  | **Continuous ZBI score  (*n* = 139)** | | **Categorical ZBI classification of  0–20 (*n* = 84) vs. 21–60 (*n* = 55)** | |
| --- | --- | --- | --- | --- |
|  | **Regression coefficient (95% CI)** | ***P* value** | **Odds ratio (95% CI)** | ***P* value** |
| Patient’s NYHA class (vs. I) | | | | |
| II | −4.10 (−9.93, 1.72) | 0.1672 | 0.81 (0.23, 2.85) | 0.7440 |
| III | −7.01 (−14.58, 0.55) | 0.0693 | 0.59 (0.12, 2.96) | 0.5220 |
| Spouse of the patient  vs. other relationship | −3.31 (−10.71, 4.08) | 0.3800 | 0.30 (0.06, 1.39) | 0.1239 |
| Female vs. male | −0.91 (−7.24, 5.42) | 0.7778 | 1.17 (0.30, 4.55) | 0.8218 |
| Lives with the patient,  yes vs. no | 2.38 (−5.99, 10.74) | 0.5779 | 2.38 (0.39, 14.43) | 0.3446 |
| Hours per week spent with the patient | −0.01 (−0.04, 0.03) | 0.6648 | 1.00 (0.99, 1.01) | 0.8759 |
| Years spent providing care to the patient | 0.01 (−0.19, 0.20) | 0.9326 | 1.00 (0.95, 1.04) | 0.8683 |
| Patient’s KCCQ-OS score | −0.28 (−0.41, −0.15) | <0.0001 | 0.96 (0.93, 0.99) | 0.0023 |
| Patient’s HADS-A score | 0.38 (−0.24, 1.01) | 0.2308 | 1.10 (0.97, 1.26) | 0.1347 |
| Patient’s HADS-D score | −0.12 (−0.78, 0.53) | 0.7123 | 0.97 (0.85, 1.10) | 0.6155 |
| CI, confidence interval; HADS-A, Hospital and Depression Scale anxiety; HADS-D, Hospital Anxiety and Depression Scale depression; KCCQ, Kansas City Cardiomyopathy Questionnaire; OS, Overall Summary; NYHA, New York Heart Association; ZBI, Zarit Burden Interview. | | | | |

**SUPPLEMENTARY TABLE S7. Moderator analysis on the relationship between patient’s continuous KCCQ-OS score and caregiver’s continuous ZBI score.**

| **Moderator** | **Model** | **Variable** | **Regression coefficient (95% CI)** | ***P* value** |
| --- | --- | --- | --- | --- |
| None | Patient’s KCCQ-OS score (alone) | - | −0.26 (–0.33, −0.20) | <0.0001 |
| **Patient-reported items as moderators** | | | | |
| Patient’s HADS-A score (continuous) | HADS-A | KCCQ-OS | −0.22 (−0.31, −0.13) | <0.0001 |
|  |  | HADS-A | 0.41 (−0.10, 0.93) | 0.1131 |
|  | HADS-A interaction with KCCQ-OS | KCCQ-OS | −0.21 (−0.33, −0.09) | 0.0008 |
|  |  | HADS-A | 0.48 (−0.42, 1.39) | 0.2934 |
|  |  | interaction | −0.00 (−0.02, 0.01) | 0.8504 |
| Patient’s HADS-D score (continuous) | HADS-D | KCCQ-OS | −0.23 (−0.33, −0.13) | <0.0001 |
|  |  | HADS-D | 0.27 (−0.25, 0.79) | 0.3024 |
|  | HADS-D interaction with KCCQ-OS | KCCQ-OS | −0.18 (−0.32, −0.04) | 0.0105 |
|  |  | HADS-D | 0.62 (−0.33, 1.56) | 0.2020 |
|  |  | interaction | −0.01 (−0.02, 0.01) | 0.3931 |
| Patient’s PROMIS Fatigue  T score (continuous) | PROMIS Fatigue | KCCQ-OS | −0.16 (−0.27, −0.04) | 0.0064 |
|  |  | PROMIS Fatigue | 0.34 (0.05, 0.63) | 0.0202 |
|  | PROMIS Fatigue interaction with KCCQ-OS | KCCQ-OS | −0.20 (−0.59, 0.18) | 0.3024 |
|  |  | PROMIS Fatigue | 0.29 (−0.21, 0.79) | 0.2474 |
|  |  | interaction | 0.00 (−0.01, 0.01) | 0.8072 |
| Patient’s SF-12 PCS score (continuous) | SF-12 PCS | KCCQ-OS | −0.29 (−0.40, −0.18) | <0.0001 |
|  |  | SF-12 PCS | 0.09 (−0.18, 0.35) | 0.5237 |
|  | SF-12 PCS with KCCQ-OS | KCCQ-OS | −0.34 (−0.61, −0.07) | 0.0151 |
|  |  | SF-12 PCS | −0.00 (−0.56, 0.55) | 0.9867 |
|  |  | interaction | 0.00 (−0.01, 0.01) | 0.7171 |
| Patient’s SF-12 MCS score (continuous) | SF-12 MCS | KCCQ-OS | −0.20 (−0.30, −0.11) | <0.0001 |
|  |  | SF-12 MCS | −0.19 (−0.39, 0.01) | 0.0590 |
|  | SF-12 MCS with KCCQ-OS | KCCQ-OS | −0.22 (−0.49, 0.05) | 0.1078 |
|  |  | SF-12 MCS | −0.21 (−0.59, 0.16) | 0.2596 |
|  |  | interaction | 0.00 (−0.01, 0.01) | 0.8829 |
| Patient’s NYHA class (I vs. II and I vs. III) | NYHA class | KCCQ-OS | −0.29 (−0.37, −0.21) | <0.0001 |
|  |  | NYHA class | −2.00 (−5.13, 1.14) | 0.2100 |
|  | NYHA class with KCCQ-OS | KCCQ-OS | −0.20 (−0.46, 0.07) | 0.1462 |
|  |  | NYHA class | 0.73 (−7.33, 8.80) | 0.8575 |
|  |  | interaction | −0.04 (−0.16, 0.07) | 0.4688 |
| Severity of muscle weakness in the arms and legs, in the last 7 days (increasing from 0 to 10) | Muscle weakness | KCCQ-OS | −0.14 (−0.23, −0.05) | 0.0019 |
|  |  | Muscle weakness | 1.51 (0.82, 2.20) | <0.0001 |
|  | Muscle weakness with KCCQ-OS | KCCQ-OS | −0.15 (−0.27, −0.02) | 0.0207 |
|  |  | Muscle weakness | 1.41 (0.00, 2.82) | 0.0505 |
|  |  | interaction | 0.00 (−0.02, 0.02) | 0.8683 |
| Severity of loss of sensation in legs or arms, in the last 7 days (increasing from 0 to 10) | Loss of sensation in legs or arms | KCCQ-OS | −0.22 (−0.29, −0.14) | <0.0001 |
|  |  | Loss of sensation in legs or arms | 0.90 (0.22, 1.58) | 0.0100 |
|  | Loss of sensation in legs or arms with KCCQ-OS | KCCQ-OS | −0.22 (−0.31, −0.13) | <0.0001 |
|  |  | Loss of sensation in legs or arms | 0.83 (−0.46, 2.12) | 0.2047 |
|  |  | interaction | 0.00 (−0.02, 0.03) | 0.9084 |
| Severity of pain, numbness, and tingling in the hand and arm, in the last 7 days (increasing from 0 to 10) | Pain, numbness, and tingling in the hand and arm | KCCQ-OS | −0.23 (−0.30, −0.15) | <0.0001 |
|  |  | Pain, numbness, and tingling in the hand and arm | 0.74 (0.10, 1.37) | 0.0230 |
|  | Pain, numbness, and tingling in the hand and arm with KCCQ-OS | KCCQ-OS | −0.23 (−0.34, −0.11) | <0.0001 |
|  |  | Pain, numbness, and tingling in the hand and arm | 0.76 (−0.68, 2.20) | 0.3002 |
|  |  | interaction | 0.00 (−0.02, 0.02) | 0.9693 |
| Severity of insomnia in the last 7 days (increasing from 0 to 10) | Insomnia | KCCQ-OS | −0.25 (−0.32, −0.17) | <0.0001 |
|  |  | Insomnia | 0.24 (−0.40, 0.88) | 0.4658 |
|  | Insomnia with KCCQ-OS | KCCQ-OS | −0.26 (−0.36, −0.16) | <0.0001 |
|  |  | Insomnia | −0.09 (−1.43, 1.25) | 0.8924 |
|  |  | interaction | 0.01 (−0.02, 0.03) | 0.5823 |
| Severity of weight loss in the last 7 days (increasing from 0 to 10)^a^ | Weight loss | KCCQ-OS | −0.25 (−0.33, −0.18) | <0.0001 |
|  |  | Weight loss | 0.24 (−0.58, 1.06) | 0.5611 |
|  | Weight loss with KCCQ-OS | KCCQ-OS | −0.25 (−0.33, −0.17) | <0.0001 |
|  |  | Weight loss | 0.37 (−1.50, 2.23) | 0.7000 |
|  |  | interaction | 0.00 (−0.04, 0.03) | 0.8848 |
| Current leg and ankle swelling (yes or no) | Leg and ankle swelling | KCCQ-OS | −0.26 (−0.33, −0.19) | <0.0001 |
|  |  | Leg and ankle swelling | 0.16 (−3.77, 4.08) | 0.9374 |
|  | Leg and ankle swelling with  KCCQ-OS | KCCQ-OS | −0.27 (−0.36, −0.19) | <0.0001 |
|  |  | Leg and ankle swelling | −1.65 (−11.41, 8.12) | 0.7395 |
|  |  | interaction | 0.03 (−0.12, 0.19) | 0.6909 |
| Current leg pain  (yes or no) | Leg pain | KCCQ-OS | −0.25 (−0.32, −0.18) | <0.0001 |
|  |  | Leg pain | 4.63 (−0.27, 9.53) | 0.0638 |
|  | Leg pain with KCCQ-OS | KCCQ-OS | −0.25 (−0.32, −0.17) | <0.0001 |
|  |  | Leg pain | 5.96 (−6.35, 18.28) | 0.3404 |
|  |  | interaction | −0.03 (−0.25, 0.20) | 0.8159 |
| Current paresthesia  (yes or no) | Paresthesia | KCCQ-OS | −0.26 (−0.33, −0.20) | <0.0001 |
|  |  | Paresthesia | 2.78 (−2.69, 8.25) | 0.3171 |
|  | Paresthesia with KCCQ-OS | KCCQ-OS | −0.28 (−0.35, −0.21) | <0.0001 |
|  |  | Paresthesia | −6.08 (−21.78, 9.63) | 0.4461 |
|  |  | interaction | 0.14 (−0.09, 0.38) | 0.2367 |
| Current paralysis (yes or no) | Paralysis | KCCQ-OS | −0.26 (−0.33, −0.19) | <0.0001 |
|  |  | Paralysis | 17.68 (4.74, 30.61) | 0.0077 |
|  | Paralysis with KCCQ-OS | KCCQ-OS | −0.26 (−0.32, −0.19) | <0.0001 |
|  |  | Paralysis | 42.41 (−0.23, 85.06) | 0.0512 |
|  |  | interaction | −0.47 (−1.25, 0.30) | 0.2313 |
| Current speech dysfunction (yes or no) | Speech dysfunction | KCCQ-OS | −0.27 (−0.34, −0.20) | <0.0001 |
|  |  | Speech dysfunction | −2.84 (−16.08, 10.40) | 0.6730 |
|  | Speech dysfunction with KCCQ-OS | KCCQ-OS | −0.26 (−0.33, −0.19) | <0.0001 |
|  |  | Speech dysfunction | 3.17 (−20.29, 26.64) | 0.7898 |
|  |  | interaction | −0.14 (−0.58, 0.31) | 0.5408 |
| Current erectile dysfunction (yes or no) | Erectile dysfunction | KCCQ-OS | −0.26 (−0.33, −0.20) | <0.0001 |
|  |  | Erectile dysfunction | 0.25 (−4.55, 5.06) | 0.9178 |
|  | Erectile dysfunction with KCCQ-OS | KCCQ-OS | −0.24 (−0.31, −0.16) | <0.0001 |
|  |  | Erectile dysfunction | 9.60 (−2.38, 21.57) | 0.1155 |
|  |  | interaction | −0.16 (−0.34, 0.03) | 0.0947 |
| Current fatigue (yes or no) | Fatigue | KCCQ-OS | −0.26 (−0.32, −0.19) | <0.0001 |
|  |  | Fatigue | 2.65 (−0.85, 6.16) | 0.1372 |
|  | Fatigue with KCCQ-OS | KCCQ-OS | −0.25 (−0.34, −0.16) | <0.0001 |
|  |  | Fatigue | 3.76 (−5.62, 13.14) | 0.4297 |
|  |  | interaction | −0.02 (−0.16, 0.12) | 0.8015 |
| Current heart failure (yes or no) | Heart failure | KCCQ-OS | −0.25 (−0.32, −0.18) | <0.0001 |
|  |  | Heart failure | 4.01 (0.14, 7.88) | 0.0424 |
|  | Heart failure with KCCQ-OS | KCCQ-OS | −0.26 (−0.42, −0.11) | 0.0008 |
|  |  | Heart failure | 2.75 (−9.90, 15.39) | 0.6687 |
|  |  | interaction | 0.02 (−0.15, 0.19) | 0.8363 |
| Current chest pain (yes or no) | Chest pain | KCCQ-OS | −0.27 (−0.33, −0.20) | <0.0001 |
|  |  | Chest pain | −2.40 (−11.81, 7.00) | 0.6150 |
|  | Chest pain with KCCQ-OS | KCCQ-OS | −0.27 (−0.34, −0.20) | <0.0001 |
|  |  | Chest pain | −4.14 (−25.27, 17.00) | 0.6997 |
|  |  | interaction | 0.03 (−0.31, 0.37) | 0.8565 |
| Current heart palpitations/irregular or rapid heartbeat not due to atrial fibrillation (yes or no) | Heart palpitations/irregular or rapid heartbeat | KCCQ-OS | −0.26 (−0.33, −0.19) | <0.0001 |
|  |  | Heart palpitations/ irregular or rapid heartbeat | 1.17 (−2.97, 5.32) | 0.5765 |
|  | Heart palpitations/irregular or rapid heartbeat with KCCQ-OS | KCCQ-OS | −0.28 (−0.36, −0.20) | <0.0001 |
|  |  | Heart palpitations/ irregular or rapid heartbeat | −3.49 (−13.95, 6.98) | 0.5120 |
|  |  | interaction | 0.08 (−0.08, 0.24) | 0.3400 |
| Current atrial fibrillation (yes or no) | Atrial fibrillation | KCCQ-OS | −0.26 (−0.33, −0.19) | <0.0001 |
|  |  | Atrial fibrillation | 1.16 (−2.26, 4.57) | 0.5051 |
|  | Atrial fibrillation with KCCQ-OS | KCCQ-OS | −0.20 (−0.29, −0.10) | 0.0001 |
|  |  | Atrial fibrillation | 9.46 (−0.01, 18.92) | 0.0502 |
|  |  | interaction | −0.13 (−0.27, 0.01) | 0.0654 |
| Current GI or urinary problems (yes or no) | GI or urinary problems | KCCQ-OS | −0.26 (−0.33, −0.19) | <0.0001 |
|  |  | GI or urinary problems | 1.24 (−3.23, 5.72) | 0.5846 |
|  | GI or urinary problems with  KCCQ-OS | KCCQ-OS | −0.24 (−0.32, −0.16) | <0.0001 |
|  |  | GI or urinary problems | 5.13 (−5.05, 15.31) | 0.3217 |
|  |  | interaction | −0.07 (−0.23, 0.09) | 0.4028 |
| **Caregiver-reported items as moderators** | | | | |
| Caregiver’s  HADS-A score (continuous) | HADS-A | KCCQ-OS | −0.17 (−0.24, −0.11) | <0.0001 |
|  |  | HADS-A | 1.45 (1.08, 1.81) | <0.0001 |
|  | HADS-A with KCCQ-OS | KCCQ-OS | −0.16 (−0.28, −0.05) | 0.0060 |
|  |  | HADS-A | 1.55 (0.55, 2.56) | 0.0027 |
|  |  | interaction | −0.00 (−0.02, 0.01) | 0.8204 |
| Caregiver’s  HADS-D score (continuous) | HADS-D | KCCQ-OS | −0.17 (−0.23, −0.11) | <0.0001 |
|  |  | HADS-D | 1.55 (1.19, 1.90) | <0.0001 |
|  | HADS-D with KCCQ-OS | KCCQ-OS | −0.19 (−0.28, −0.09) | <0.0001 |
|  |  | HADS-D | 1.32 (0.41, 2.22) | 0.0045 |
|  |  | interaction | 0.00 (−0.01, 0.02) | 0.5814 |
| Caregiver’s PROMIS Fatigue  T score (continuous) | PROMIS Fatigue | KCCQ-OS | −0.15 (−0.21, −0.09) | <0.0001 |
|  |  | PROMIS Fatigue | 0.78 (0.64, 0.93) | <0.0001 |
|  | PROMIS Fatigue interaction with KCCQ-OS | KCCQ-OS | −0.19 (−0.47, 0.08) | 0.1715 |
|  |  | PROMIS Fatigue | 0.73 (0.33, 1.12) | 0.0004 |
|  |  | interaction | 0.00 (0.00, 0.01) | 0.7535 |
| Caregiver’s SF-12 PCS score (continuous) | SF-12 PCS | KCCQ-OS | −0.27 (−0.33, −0.20) | <0.0001 |
|  |  | SF-12 PCS | −0.26 (−0.43, −0.09) | 0.0029 |
|  | SF-12 PCS with KCCQ-OS | KCCQ-OS | 0.14 (−0.21, 0.48) | 0.4369 |
|  |  | SF-12 PCS | 0.26 (−0.20, 0.72) | 0.2721 |
|  |  | interaction | −0.01 (−0.02, −0.00) | 0.0193 |
| Caregiver’s SF-12 MCS score (continuous) | SF-12 MCS | KCCQ-OS | −0.16 (−0.22, −0.10) | <0.0001 |
|  |  | SF-12 MCS | −0.69 (−0.83, −0.54) | <0.0001 |
|  | SF-12 MCS with KCCQ-OS | KCCQ-OS | −0.19 (−0.46, 0.08) | 0.1661 |
|  |  | SF-12 MCS | −0.73 (−1.09, −0.36) | 0.0001 |
|  |  | interaction | 0.00 (0.00. 0.01) | 0.8120 |
| Caregiver’s age (continuous) | Age | KCCQ-OS | −0.26 (−0.33, −0.19) | <0.0001 |
|  |  | Age | −0.06 (−0.19, 0.80) | 0.3826 |
|  | Age with KCCQ-OS | KCCQ-OS | −0.35 (–0.73, 0.03) | 0.0696 |
|  |  | Age | −0.15 (−0.55, 0.26) | 0.4714 |
|  |  | interaction | 0.00 (0.00, 0.01) | 0.6484 |
| Caregiver’s relationship with patient (spouse vs. other) | Relationship category | KCCQ-OS | −0.26 (−0.33, −0.19) | <0.0001 |
|  |  | Relationship | 0.86 (−0.64, 2.37) | 0.2600 |
|  | Relationship with KCCQ-OS | KCCQ-OS | −0.19 (−0.40, 0.01) | 0.0628 |
|  |  | Relationship | 2.34 (−2.21, 6.89) | 0.3116 |
|  |  | interaction | −0.02 (−0.09, 0.04) | 0.4980 |
| Caregiver’s sex (male vs. female) | Sex | KCCQ-OS | −0.26 (−0.33, −0.20) | <0.0001 |
|  |  | Sex | 0.00 (−4.84, 4.84) | 1.0000 |
|  | Sex with KCCQ-OS | KCCQ-OS | −0.13 (−0.38, 0.12) | 0.3039 |
|  |  | Sex | 8.54 (−7.52, 24.60) | 0.2954 |
|  |  | interaction | −0.12, (−0.34, 0.10) | 0.2726 |
| Hours per week caregiver spends with patient (continuous) | Hours per week | KCCQ-OS | −0.26 (−0.33, −0.19) | <0.0001 |
|  |  | Hours per week | −0.01 (−0.03, 0.02) | 0.5292 |
|  | Hours per week with KCCQ-OS | KCCQ-OS | −0.23 (−0.34, −0.12) | <0.0001 |
|  |  | Hours per week | 0.01 (−0.06, 0.08) | 0.7022 |
|  |  | interaction | −0.00 (0.00, 0.00) | 0.5210 |
| Years caregivers has provided care (continuous) | Years | KCCQ-OS | −0.28 (−0.35, −0.20) | <0.0001 |
|  |  | Years | 0.01 (−0.19, 0.21) | 0.9367 |
|  | Years with KCCQ-OS | KCCQ-OS | −0.29 (−0.37, −0.20) | <0.0001 |
|  |  | Years | −0.27 (−1.26, 0.72) | 0.5878 |
|  |  | interaction | 0.00 (−0.01, 0.02) | 0.5690 |
| Caregiver and patient live together (yes vs. no) | Live together | KCCQ-OS | −0.26 (−0.33, −0.19) | <0.0001 |
|  |  | Live together | 1.38 (−2.21, 4.96) | 0.4494 |
|  | Live together with KCCQ-OS | KCCQ-OS | −0.24 (−0.45, −0.04) | 0.0208 |
|  |  | Live together | 2.24 (−7.50, 11.98) | 0.6508 |
|  |  | interaction | −0.01 (−0.16, 0.13) | 0.8514 |
| Caregiver required to help the patient with bathing (no vs. yes) | Bathing help | KCCQ-OS | −0.26 (−0.33, −0.18) | <0.0001 |
|  |  | Bathing help | 1.27 (−2.91, 5.44) | 0.5501 |
|  | Bathing help with KCCQ-OS | KCCQ-OS | −0.25 (−0.49, −0.02) | 0.0362 |
|  |  | Bathing help | 1.39 (−7.87, 10.65) | 0.7671 |
|  |  | interaction | 0.00 (−0.20, 0.19) | 0.9760 |
| Caregiver required to help the patient with cooking (no vs. yes) | Cooking help | KCCQ-OS | −0.27 (−0.34, −0.19) | <0.0001 |
|  |  | Cooking help | 0.22 (−2.57, 3.02) | 0.8745 |
|  | Cooking help with KCCQ-OS | KCCQ-OS | −0.33 (−0.50, −0.17) | 0.0001 |
|  |  | Cooking help | −2.33 (−8.59, 3.92) | 0.4629 |
|  |  | interaction | 0.05 (−0.06, 0.16) | 0.3687 |
| Caregiver required to help the patient with cleaning (no vs. yes) | Cleaning help | KCCQ-OS | −0.25 (−0.33, −0.17) | <0.0001 |
|  |  | Cleaning help | 1.05 (−1.75, 3.86) | 0.4606 |
|  | Cleaning help with KCCQ-OS | KCCQ-OS | −0.26 (−0.44, −0.08) | 0.0038 |
|  |  | Cleaning help | 0.78 (−5.26, 6.82) | 0.7994 |
|  |  | interaction | 0.01 (−0.11, 0.12) | 0.9203 |
| Caregiver required to help the patient getting on/off the toilet (no vs. yes) | Help getting on/off the toilet | KCCQ-OS | −0.27 (−0.34, −0.20) | <0.0001 |
|  |  | Help getting on/off the toilet | −2.60 (−7.71, 2.51) | 0.3160 |
|  | Help getting on/off the toilet with KCCQ-OS | KCCQ-OS | −0.26 (−0.55, 0.03) | 0.0777 |
|  |  | Help getting on/off the toilet | −2.05 (−15.38, 11.28) | 0.7618 |
|  |  | interaction | −0.01 (−0.28, 0.26) | 0.9295 |
| Caregiver required to help the patient getting in/out of bed (no vs. yes) | Help getting in/out of bed | KCCQ-OS | −0.27 (−0.34, −0.20) | <0.0001 |
|  |  | Help getting in/out of bed | −2.73 (−7.76, 2.31) | 0.2865 |
|  | Help getting in/out of bed with KCCQ-OS | KCCQ-OS | −0.27 (−0.54, 0.00) | 0.0491 |
|  |  | Help getting in/out of bed | −2.69 (−14.81, 9.43) | 0.6622 |
|  |  | interaction | 0.00 (−0.25, 0.25) | 0.9944 |
| Caregiver required to help the patient walk (no vs. yes) | Walking help | KCCQ-OS | −0.26 (−0.34, −0.19) | <0.0001 |
|  |  | Walking help | 0.30 (−3.76, 4.36) | 0.8850 |
|  | Walking help with KCCQ-OS | KCCQ-OS | −0.25 (−0.49, −0.01) | 0.0437 |
|  |  | Walking help | 0.89 (−8.91, 10.69) | 0.8577 |
|  |  | interaction | −0.01 (−0.21, 0.19) | 0.8955 |
| Except ^a^*n* = 207; median severity, 0.0 (interquartile range, 0.0, 1.0), further detail on each item is provided in: Ponti L, Hsu K, Damy T, Villacorta E, Verheyen N, Keohane D, et al. Burden of untreated transthyretin amyloid cardiomyopathy on patients and their caregivers by disease severity: results from a multicenter, non-interventional, real-world study. *Front Cardiovasc Med* (2023) 10:1238843. doi: 10.3389/fcvm.2023.1238843  CI, confidence interval; GI, gastrointestinal; HADS-A, Hospital and Depression Scale anxiety; HADS-D, Hospital Anxiety and Depression Scale depression; KCCQ, Kansas City Cardiomyopathy Questionnaire; MCS, Mental Component Summary; NYHA, New York Heart Association; OS, Overall Summary; PCS, Physical Component Summary; PROMIS, Patient-Reported Outcomes Measurement Information System; SF-12, 12-item Short Form Health Survey; ZBI, Zarit Burden Interview. | | | | |
